# TORC1‐mediated sensing of chaperone activity alters glucose metabolism and extends lifespan

**DOI:** 10.1111/acel.12623

**Published:** 2017-06-14

**Authors:** Matea Perić, Anita Lovrić, Ana Šarić, Marina Musa, Peter Bou Dib, Marina Rudan, Andrea Nikolić, Sandra Sobočanec, Ana‐Matea Mikecin, Sven Dennerlein, Ira Milošević, Kristian Vlahoviček, Nuno Raimundo, Anita Kriško

**Affiliations:** ^1^ Mediterranean Institute for Life Sciences Mestrovicevo setaliste 45 21000 Split Croatia; ^2^ Division of Molecular Medicine Rudjer Boskovic Institute Bijenicka 54 10000 Zagreb Croatia; ^3^ Institut für Zellbiochemie Universitätsmedizin Göttingen Humboldtallee 23 D‐37073 Göttingen Germany; ^4^ European Neuroscience Institute Grisebachstraße 5 37077 Göttingen Germany; ^5^ Bioinformatics Group Division of Biology Department of Molecular Biology Faculty of Science University of Zagreb Zagreb Croatia

**Keywords:** ageing, glucose starvation, mitochondria, protein chaperones, replicative lifespan, Snf1/AMPK, TORC1, yeast

## Abstract

Protein quality control mechanisms, required for normal cellular functioning, encompass multiple functions related to protein production and maintenance. However, the existence of communication between proteostasis and metabolic networks and its underlying mechanisms remain elusive. Here, we report that enhanced chaperone activity and consequent improved proteostasis are sensed by TORC1 via the activity of Hsp82. Chaperone enrichment decreases the level of Hsp82, which deactivates TORC1 and leads to activation of Snf1/AMPK, regardless of glucose availability. This mechanism culminates in the extension of yeast replicative lifespan (RLS) that is fully reliant on both TORC1 deactivation and Snf1/AMPK activation. Specifically, we identify oxygen consumption increase as the downstream effect of Snf1 activation responsible for the entire RLS extension. Our results set a novel paradigm for the role of proteostasis in aging: modulation of the misfolded protein level can affect cellular metabolic features as well as mitochondrial activity and consequently modify lifespan. The described mechanism is expected to open new avenues for research of aging and age‐related diseases.

## Introduction

Protein homeostasis (proteostasis) encompasses the equilibrium between synthesis, conformational maintenance, and degradation of damaged proteins. While protein synthesis has a critical role in making the proteome and promoting cell growth, failure to eliminate misfolded proteins can lead to inactivation of functional proteins as well as cell degeneration and death. Cellular proteostasis is maintained by a network of molecular chaperones, protein degradation machineries, and stress–response pathways, whose coordinated action senses and counteracts protein misfolding (Wolff *et al*., 2014). Molecular chaperones, including the heat‐shock proteins (HSPs), are ubiquitously present cellular proteins, which display a wide spectrum of folding‐oriented activities, coping with regular protein folding events, as well as stress‐induced protein misfolding (Morano *et al*., 1998). The efficiency of proteostasis may decline, with well‐described consequences, especially in the context of numerous diseases and aging (Labbadia & Morimoto, 2015). As a result of such a decline, proteins cannot maintain their native fold or perform their function and, consequently, cells promote the removal of damaged proteins through their degradation, refolding, and/or aggregation (Brandvold & Morimoto, 2015). On the other hand, the research on phenotypes related to the alleviation of protein misfolding is not keeping pace. The improvement of disaggregase activity in yeast was found to diminish the accumulation of insoluble aggregates during aging and restored degradation of 26S proteasome substrates in aged cells (Andersson *et al*., 2013). Because of extensive interest, the cell‐wide response to the enrichment in chaperone activity has not received any attention.

Despite the indisputable importance of cellular proteostasis on multiple levels, the relationships between proteostasis and other cellular pathways remain poorly understood. While signaling networks have evolved to convey information on any changes among themselves and turn them into critical decisions regarding cellular destiny, little is known about the role of molecular chaperones in the regulation of metabolic networks (Rowland *et al*., 2012).

Here, we investigate the cellular response to improved proteostasis resulting from enhanced chaperone activity and how it communicates with the metabolic networks, using budding yeast as a model system. We show that the response to protein misfolding alleviation is a phenocopy of the glucose starvation response. This outcome relies on deactivation of TORC1 and consequent activation of the Snf1 kinase, a yeast ortholog of mammalian AMPK. Further, our results reveal the role of Tor1 kinase as a sensor of improved chaperone function and protein folding environment. Remarkably, all mutants characterized by improved proteostasis display an extension of the replicative lifespan (RLS). We identify the increase in cellular respiratory activity as the trigger of the RLS extension, dependent on TORC1 deactivation and Snf1 activation, thus demonstrating that the observed lifespan extension fully relies on the metabolic changes originating from proteostasis improvement. The data presented here demonstrate that, in the context of normal cellular functioning, as well as aging, the role of protein chaperones goes beyond the management of misfolded proteins, with downstream effects on cellular metabolism and mitochondrial network.

## Results

### Differential gene expression in chaperone‐enriched strains reveals a phenocopy of glucose starvation

**Figure 1:**
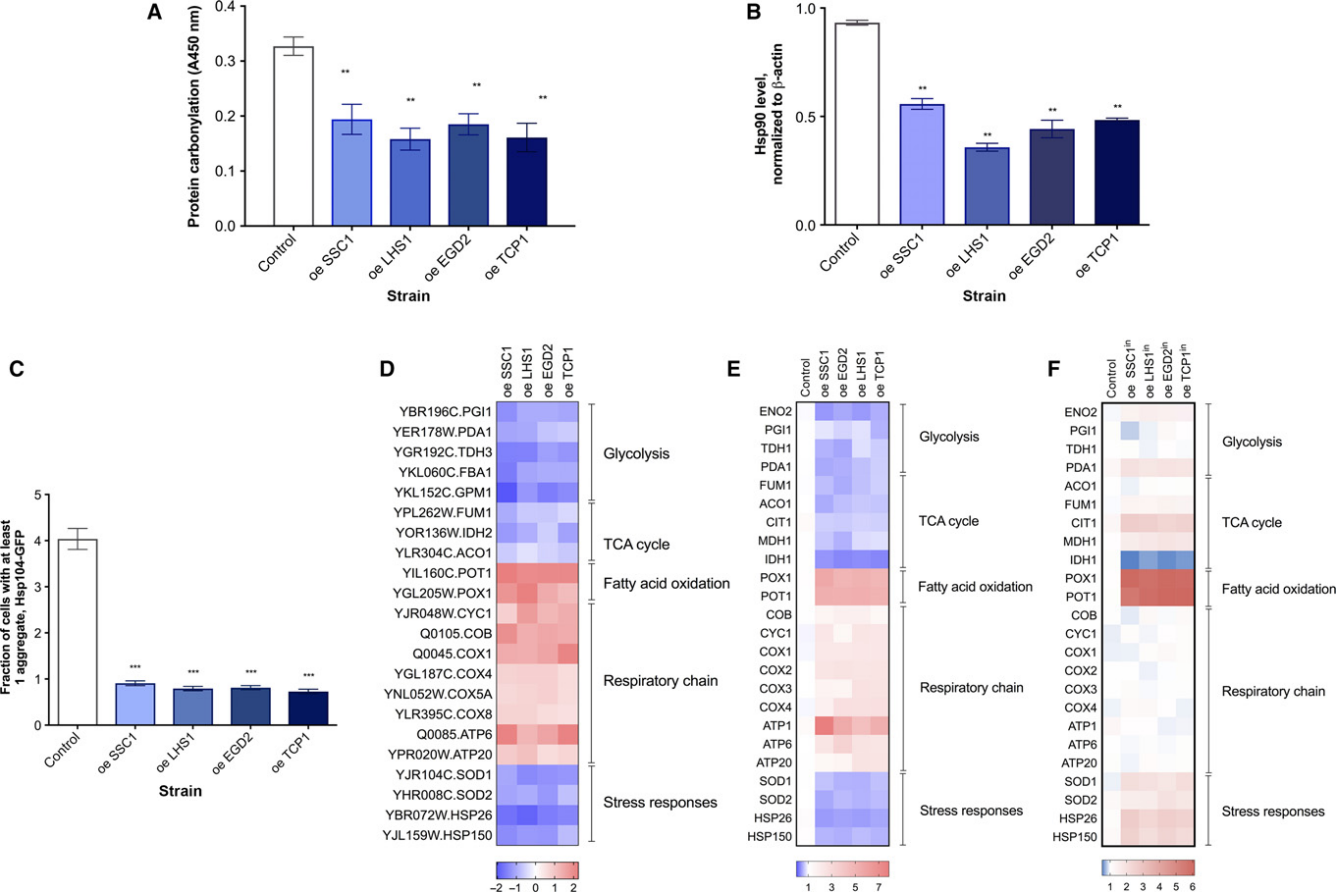
Enrichment in chaperone activity alleviates protein damage and activates a glucose starvation‐like response. (A) Protein carbonylation (PC) is decreased in the ChES. As a control, wild‐type yeast with an empty vector was used. Data on the graph are mean ± SD from at least three independent cultures, each performed in technical triplicate. *P* values were calculated using ANOVA plus post hoc. ***P < 0.001; **P < 0.01; *P < 0.05. (B) Hsp82 levels are decreased in ChES. Hsp82‐specific band intensity was normalized to the intensity of β‐actin (loading control). Control is wild‐type yeast with an empty vector. Data on the graph are mean ± SD from at least three independent cultures, each performed in technical triplicate. *P* values were calculated using ANOVA plus post hoc. ***P < 0.001; **P < 0.01; *P < 0.05. (C) ChESs are characterized by decreased protein aggregation propensity. Protein aggregation propensity is expressed as the fraction of cells with at least one aggregate. More than 1000 cells were screened for aggregates starting from two independent exponential yeast cultures for each strain. Control is wild‐type yeast with an empty vector. Data on the graph are mean ± SD from two independent cultures. *P* values were calculated using ANOVA plus post hoc. ***P < 0.001; **P < 0.01; *P < 0.05. (D) Differential gene expression in ChES reveals a glucose starvation‐like response. Heat map displays RNASeq‐derived differential expression (contrasted to control) of selected genes, grouped according to their respective metabolic pathways. Gray X signs indicate statistically insignificant fold changes. Gene names are indicated on the left of each heat map. (E) qPCR measurement of differential gene expression confirms a glucose starvation response phenocopy in ChES. Color of the squares on the heat map corresponds to the mean value of the log fold change from three biological and three technical replicates. UBC6 was used for normalization. Control is wild‐type yeast with an empty vector. (F) Enrichment in inactivated versions of each chaperone (iChES) does not phenocopy glucose starvation response. Color of the squares on the heat map corresponds to the mean value of the log fold change from three biological and three technical replicates. UBC6 was used for normalization. Control is wild‐type yeast with an empty vector.

To decrease the amount of misfolded proteins, we independently overexpressed protein chaperones located in different cellular compartments—nascent polypeptide‐associated complex (NAC, *EGD2*), cytosolic T‐complex (*TCP1*), mitochondrial HSP70 (*SSC1*), and HSP70 family chaperone from the endoplasmic reticulum (*LHS1*). For brevity, we termed these mutants chaperone‐enriched strains (ChESs). The level of enrichment with each chaperone was estimated by flow cytometry. We find the Egd2, Lhs1, and Tcp1 enriched threefold, while Ssc1 is enriched 2.5‐fold (Fig. S1A, Supporting information). We next evaluated whether such enrichment in chaperone function results in decrease in misfolded protein amount. ChESs display, on average, a 40% decrease in protein carbonylation (PC) level (Fig. 1A). Even though PC has long been considered as a marker of oxidative stress, it has been shown that misfolded proteins and proteins characterized by decreased conformational stability are more prone to PC (Vidovic *et al*., 2014), rendering it an indicator of protein folding quality. Moreover, a strong downregulation of the Hsp90 chaperone is observed, relative to the control (Figs 1B and S1B, Supporting information). In addition, the level of Hsp104‐dependent protein aggregation is decreased in ChES from ~4% of cells with aggregates in the control strain to ~1% in ChES (Figs 1C and S2, Supporting information). Together, these observations suggest that the chaperone enrichment indeed led to a decrease in the extent of protein misfolding.

As an additional control, we have enriched the cells with the inactivated version of each chaperone (iChES, Fig. S3, Supporting information, for details, see Experimental procedures) and detected a subtle increase in Hsp104‐dependent protein aggregation from 4% of cells with aggregates to 7–8% (Figs S1C and S2, Supporting information). This increase in cellular fraction containing aggregates was accompanied by an average of 15% increase in protein carbonylation level (Fig. S1D, Supporting information).

With the aim of uncovering general trends in gene expression in ChES, we applied RNA sequencing (RNASeq) (results summarized in Table S1, Supporting information). The most striking results are the downregulation of key glycolytic and TCA cycle enzymes, strongly arguing toward the repression of glycolysis and the TCA cycle (Fig. 1D). The increased expression levels of the respiratory chain subunits suggest an increase in respiration in ChES (Fig. 1D). Additionally, strong upregulation of *POX1* (encoding fatty‐acyl coenzyme A oxidase) and *POT1* (encoding 3‐ketoacyl‐CoA thiolase) in ChES brings forward fatty acids as the preferred energy source (Fig. 1D). The results obtained by RNASeq were confirmed by qPCR, where target genes display similar trends as observed by RNASeq (Fig. 1E). Clearly, differential gene expression in ChES displays a glucose starvation‐like response (Lin *et al*., 2002).

Moreover, several genes typically upregulated during the heat‐shock response (e.g., Hsp90, Hsp150, and Hsp26) are downregulated in ChES, indicating a decrease in proteotoxic stress to below the basal levels observed in the control strain (Fig. 1D, Table S1, Supporting information). Furthermore, *SOD1*, cytosolic copper–zinc superoxide dismutase, and *SOD2*, mitochondrial manganese superoxide dismutase, are both significantly downregulated in ChES relative to the control, suggesting an overall decrease in the reactive oxygen species (ROS) level (Table S1, Supporting information). To confirm this observation, we monitored fluorescence that results from the reaction of 2′,7′‐dichlorodihydrofluorescein (H2DCF) with intracellular ROS, using a FACS‐based procedure. ChES mutants showed an average of 40% decrease in ROS levels compared to the control strain (Fig. S1E, Supporting information).

The iChES strains (Fig. S3, Supporting information), enriched in the inactivated version of each chaperone, do not exhibit a decrease in glycolytic and TCA cycle enzymes’ expression level, or an increase in the respiratory chain component expression (Figs 1F and S1F, Supporting information). These results suggest that the differential gene expression pattern detected in ChES is reliant on increased chaperone activity and/or the beneficial consequences that it has on protein folding environment. However, strong upregulation of *POT1* and *POX1*, observed also in iChES (Figs 1F and S1F, Supporting information), suggests that the increase in the fatty acid oxidation is not related to the increased chaperone activity in ChES. The mechanism and the role of increased fatty acid oxidation in ChES and iChES remain unclear based on the presented data and will be the subject of future research.

### ChESs are characterized by increased oxygen consumption

**Figure 2:**
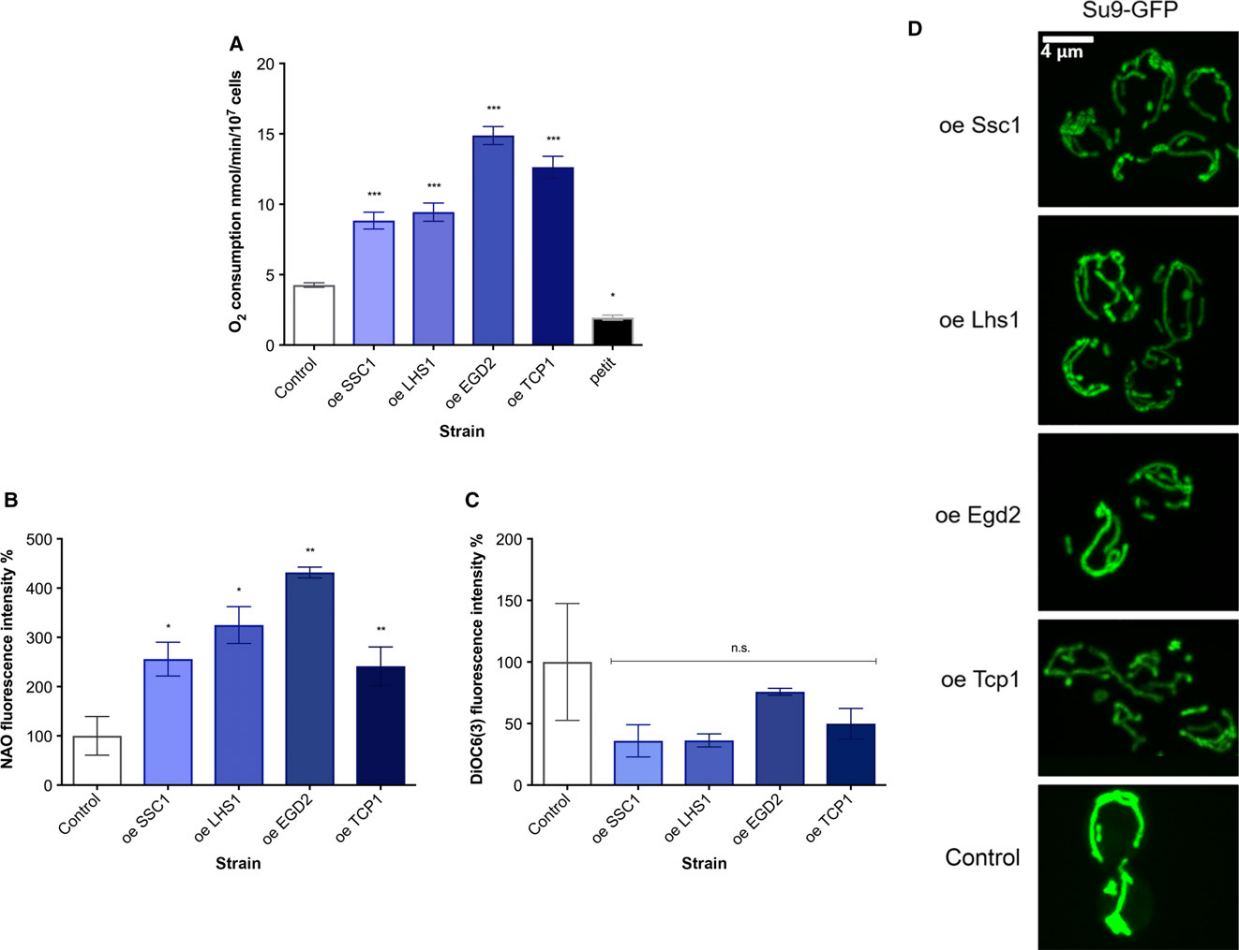
Enrichment in chaperone activity is accompanied by increased oxygen consumption and hyperfused mitochondria. (A) Oxygen consumption rates are increased in ChES. Control is wild‐type yeast with an empty vector. Data on the graph are mean ± SD from three biological and three technical replicates. *P* values were calculated using ANOVA plus post hoc. ***P < 0.001; **P < 0.01; *P < 0.05. (B) Mitochondrial biomass is increased in ChES. Data are mean ± SD from at least three independent cultures, each performed in triplicate. ***P < 0.001; **P < 0.01; *P < 0.05 (ANOVA plus post hoc). (C) Mitochondrial membrane potential‐related fluorescence via DiOC6(3) is unchanged. Data are mean ± SD from at least three independent cultures, each performed in triplicate. ***P < 0.001; **P < 0.01; *P < 0.05 (ANOVA plus post hoc). (D) Mitochondria display an increase in volume. More than 300 cells from two biological replicates were inspected, and the images here display representative examples.

We further explored the glucose starvation‐like phenotype suggested by the ChES transcriptome. We first measured O_2_ consumption in ChES, mimicking flask culture conditions (30 °C temperature, with stirring). The growth curves of studied strains with the labeled point at which we have harvested the cells are shown in Fig. S4A (Supporting information). All of the ChESs exhibited, on average, a twofold increase in O_2_ consumption in comparison with the control strain, with most prominent change observed in *EGD2* overexpression followed by *TCP1*,* LHS1,* and *SSC1* (Fig. 2A). Petite strain, which has no functional electron transport chain, was included as a negative control and showed respiration deficiency. Moreover, the iChESs, enriched in inactivated version of each chaperone, display no increase in the oxygen consumption (Fig. S4B, Supporting information), suggesting that the increase in respiratory activity in ChES stems from the activity of studied chaperones.

As elevated O_2_ consumption can be a result of enhanced mitochondrial capacity, we measured the mitochondrial mass. 10‐N‐Nonyl acridine orange (NAO) fluorescence, indicative of cardiolipin content/mitochondrial mass, displayed an increase in ChES compared to the control (Fig. 2B). On the other hand, no significant difference in fluorescence intensity was observed after 3,3′‐dihexyloxacarbocyanine iodide (DiOC6(3)) staining between any of the ChES strains and the control, reporting no difference in the inner mitochondrial membrane potential (Fig. 2C). Using MitoLoc plasmid, where GFP is fused to the fungal mitochondrial localization signal of the F_0_‐ATPase subunit 9 (preSU9) of *Neurospora crassa,* we characterized the mitochondrial network using spinning disk confocal microscopy. We found the strains to display a strong increase in mitochondrial volume (Figs 2D and S4C, Supporting information), in accordance with the increased NAO fluorescence in ChES (Fig. 2B) and increased oxygen consumption (Fig. 2A).

To further characterize the respiratory chain (RC) in ChES, mitochondria were solubilized in digitonin and subjected to the blue native PAGE analysis for the assembly state of the RC complexes, as well as for the measurement of their *in vitro* activity. We have found no significant difference between the strains indicating that the amount of RC complexes per mitochondrion is similar in ChES and control strains, and that the increased oxygen consumption results from increased mitochondrial mass (Fig. S4D, Supporting information).

### Snf1/AMPK activation is responsible for the glucose starvation phenocopy in ChES

**Figure 3:**
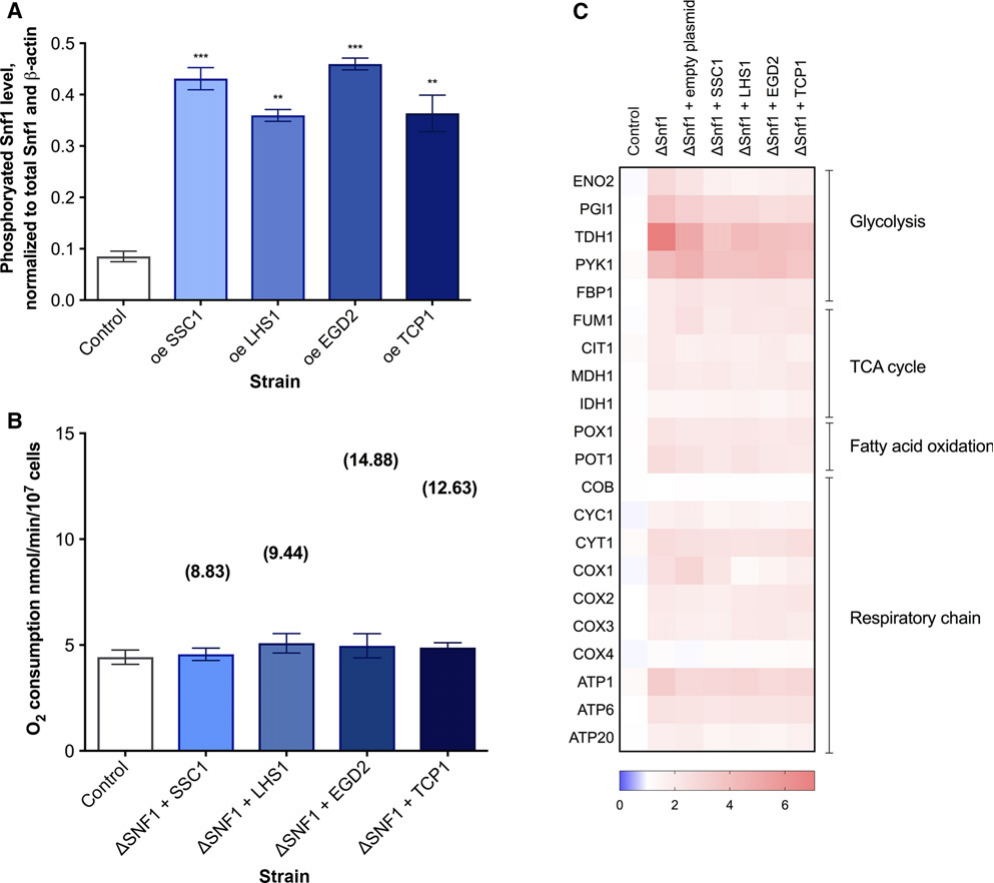
Snf1/AMPK deletion suppresses the glucose starvation‐like response in ChES. (A) The level of phosphorylated Snf1 is increased in ChES. Data represent quantification (ImageJ) of Western blot results from three independent experiments. Phospho‐Snf1 band intensity was normalized to the intensity of total Snf1 and β‐actin (loading control). Control is wild‐type yeast with an empty vector. Data are mean ± SD from three independent cultures, each performed in triplicate. ***P < 0.001; **P < 0.01; *P < 0.05 (ANOVA plus post hoc). (B) Snf1 is required to trigger the glucose starvation‐like response by enhanced chaperone activity. Transcript levels of target genes in ChES in the absence of Snf1 are compared to the control (wild‐type yeast with an empty plasmid). UBC6 was used for normalization. Color of the squares on the heat map corresponds to the mean value of the log fold change from three biological and three technical replicates. (C) Oxygen consumption of the ChES declines in the absence of Snf1. In brackets are the mean values of oxygen consumption of the corresponding ChES in the wild‐type background, added for comparison. As a control strain, the ΔSnf1 yeast with an empty vector was used. Data are mean ± SD from at least three independent cultures, each performed in triplicate. ***P < 0.001; **P < 0.01; *P < 0.05 (ANOVA plus post hoc).

Based on the literature related to the glucose starvation‐like phenotype observed in ChES, we hypothesized that this phenotype could be a result of the activation of Snf1 kinase, a major regulator of glucose metabolism in yeast (reviewed in Hedbacker & Carlson, 2009). Therefore, to estimate the Snf1 activity in the studied strains, we measured the level of phosphorylated Snf1 by Western blot and detected its increase in ChES relative to the control (Figs 3A and S5A, Supporting information).

Further, we have overexpressed each of the four chaperones in the genetic background of *Snf1* deletion and measured the differences in the related gene expression patterns by qPCR (Figs 3B and S5B, Supporting information, compared with Fig. 1E). The absence of Snf1 itself yields numerous changes in gene expression: a significant upregulation of glycolytic and TCA cycle enzymes, as well as OxPhos complexes (Figs 3C and S5B, Supporting information). The presence of the empty plasmid in the *Snf1*‐deficient background does not influence this expression pattern. Upon chaperone enrichment in *Snf1*‐deficient background, the glucose starvation‐like response characteristic of chaperone enrichment in the WT background is now absent (Figs 3B and S5B, Supporting information). Consistently, the increase in oxygen consumption does not occur upon chaperone overexpression in the absence of Snf1 (Fig. 3C). These results strongly suggest that Snf1 kinase receives the information about the cellular proteostasis and, via its canonical activity, modulates metabolism. However, the question persists: how does the information on alleviation of protein misfolding reach Snf1 kinase, that is which cellular component is responsible for sensing the folding environment and relaying the message on to the metabolic networks?

### Tor1 acts as a sensor of folding environment and a negative regulator of Snf1

**Figure 4:**
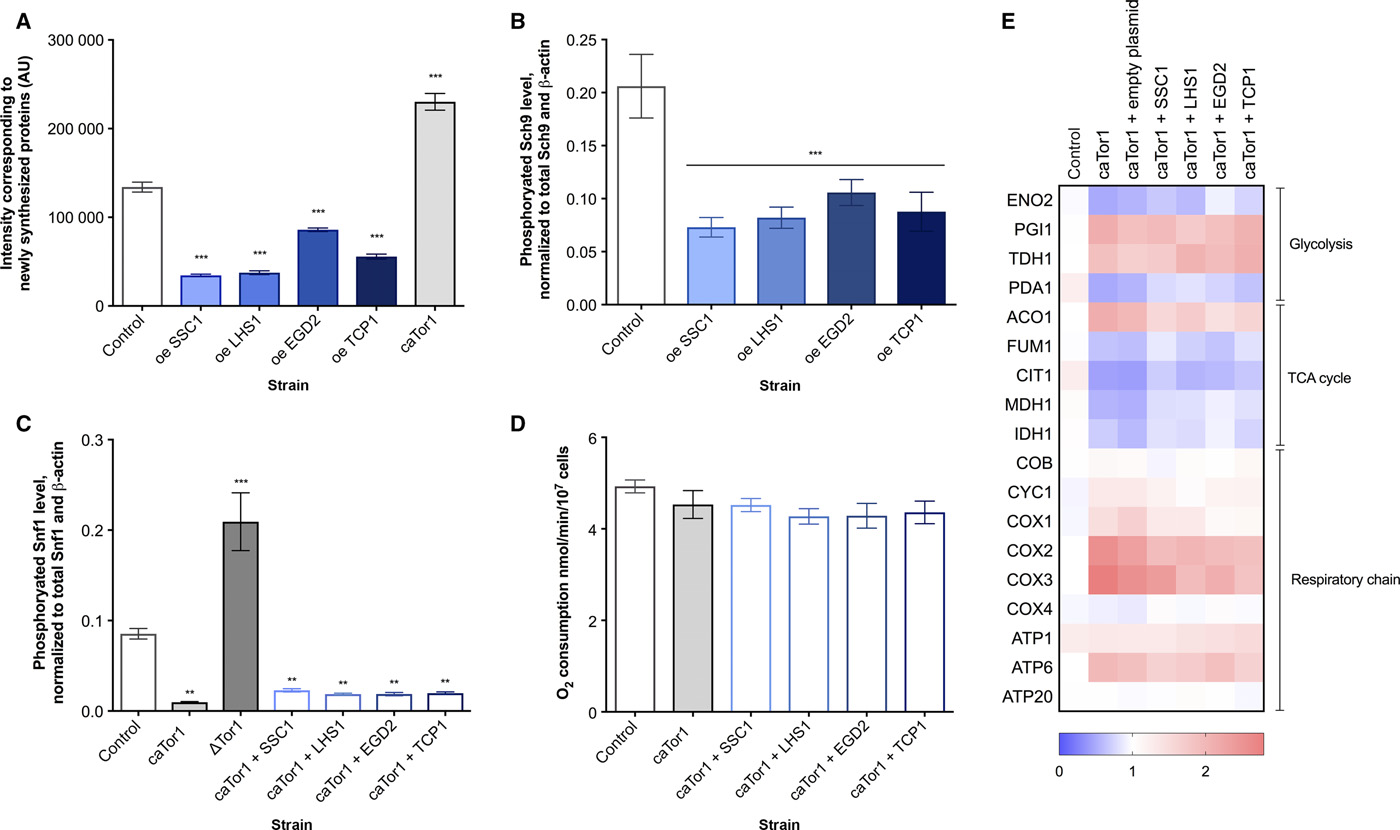
TORC1 undergoes deactivation upon chaperone enrichment and negatively regulates Snf1/AMPK. (A) The amount of newly translated proteins is decreased in ChES. Data represent quantification (ImageJ) of Western blot results from three independent experiments (mean with SD). ***P < 0.001; **P < 0.01; *P < 0.05 (ANOVA plus post hoc). (B) ChESs display a decreased level of phosphorylated Sch9. Sch9 is a downstream target of Tor1 kinase; thus, TOR deactivation is confirmed in ChES. Data represent quantification (ImageJ) of Western blot results from three independent experiments. Phospho‐Sch9 band intensity was normalized to the total level of Sch9 and to the intensity of β‐actin (loading control). Control is wild‐type yeast with an empty vector. Data are mean ± SD from at least three independent cultures, each performed in triplicate. ***P < 0.001; **P < 0.01; *P <0.05 (ANOVA plus post hoc). (C) Tor1 acts as a negative regulator of Snf1. The level of phosphorylated Snf1 is measured in the caTor1 strain and Tor1‐null strain and chaperone enrichment in the background of caTor1. Data represent quantification (ImageJ) of Western blot results from three independent experiments. Phospho‐Snf1 band intensity was normalized to the intensity of total Snf1 and β‐actin (loading control). Control is wild‐type yeast with an empty vector. Data are mean ± SD from at least three independent cultures, each performed in triplicate. ***P < 0.001; **P < 0.01; *P < 0.05 (ANOVA plus post hoc). (D) Oxygen consumption rates remain unchanged in the case of chaperone enrichment in the caTor1 background. Control is wild‐type yeast with an empty vector. Data are mean ± SD from at least three independent cultures, each performed in triplicate. ***P < 0.001; **P < 0.01; *P < 0.05 (ANOVA plus post hoc). (E) The differential gene expression (by qPCR) characteristic of chaperone enrichment in the caTor1 background does not phenocopy glucose starvation response. Color of the squares on the heat map corresponds to the mean value of the log fold change from three biological and three technical replicates. UBC6 was used for normalization. Control is wild‐type yeast with an empty vector.

Given the known interplay between TOR and Snf1, we hypothesized the potential involvement of the TORC1 complex as a mediator between the folding environment and metabolism (Su *et al*., 2016). Hence, we measured the amount of newly translated proteins within one generation time of each studied strain. The results show that the ChESs perform significantly less translation relative to the control (Figs 4A and S5C, Supporting information), suggesting a decrease in the TORC1 activity. In addition, ChESs display significantly lower levels of Sch9 phosphorylation, confirming TORC1 deactivation (Figs 4B and S5D, Supporting information). In addition, this result shows that the amount of newly translated proteins also serves as a proxy for TORC1 activity. Therefore, deactivation of TORC1 may be a critical part of the response to the chaperone enrichment, and the related alleviation of protein misfolding. To test this hypothesis, we introduced I1954V mutation into Tor1 kinase, rendering it constitutively active and unable to undergo deactivation (caTor1) (Hardt *et al*., 2011). The amount of newly translated proteins has confirmed the increase in the translation activity in the caTor1 strain (Figs 4A and S5C, Supporting information). We have further evaluated whether the activity of TORC1 affects the phosphorylation level of Snf1. Constitutive activity of Tor1 has resulted in a sharp decrease in the Snf1 phosphorylation level, reporting on a decline in its activity (Figs 4C and S5E, Supporting information). On the contrary, absence of Tor1 resulted in increased phosphorylation/activity of Snf1, compared to the control strain. While a direct interaction between Tor1 and Snf1 is not implied, these two observations indicate that Tor1 kinase may serve as a negative regulator of Snf1, in agreement with published data (Orlova *et al*., 2006). Further, the overexpression of each of the four chaperones in the caTor1 background does not result in the activation of Snf1 (Figs 4C and S5E, Supporting information). Oxygen consumption remains unchanged in strains with chaperone enrichment in the caTor1 genetic background (Fig. 4D), confirming the absence of phenotype related to Snf1 activation and identifying Tor1 deactivation as a central event responsible for Snf1 activation in ChES. This result is supported by the differential gene expression in caTor1 with chaperone enrichment showing the lack of response typical for chaperone enrichment in the control background (Figs 4E and S5F, Supporting information).

**Figure 5:**
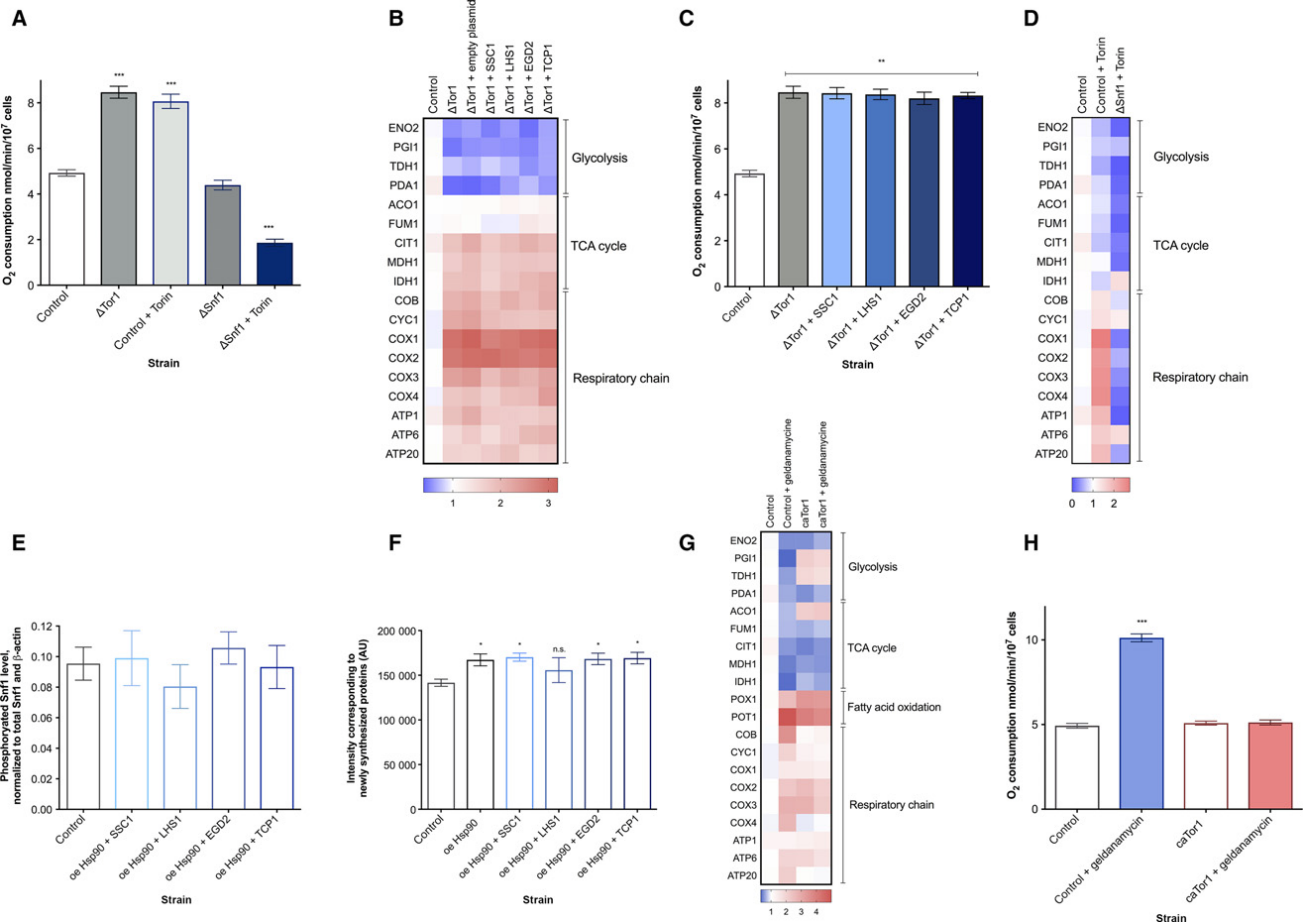
Downregulation of Hsp82 is required for the TORC1 deactivation and Snf1 activation in ChES. (A) Oxygen consumption is increased in the ΔTor1 strain; however, TORC1 deactivation of ΔSnf1 strain causes a strong respiration decline. Control is wild‐type yeast with an empty vector. Data are mean ± SD from at least three independent cultures, each performed in triplicate. ***P < 0.001; **P < 0.01; *P < 0.05 (ANOVA plus post hoc). (B) Differential gene expression in ΔTor1 strain shows a phenocopy of the glucose starvation response. The detected response is consistent with the one found in ChES and is consistent with increased Snf1 activation. Chaperone enrichment of the ΔTor1 background does not lead to any further changes in the gene expression pattern. Color of the squares on the heat map corresponds to the mean value of the log fold change from three biological and three technical replicates. UBC6 was used for normalization. Control is wild‐type yeast with an empty vector. (C) Oxygen consumption does not change upon chaperone enrichment in the ΔTor1 background. Control is wild‐type yeast with an empty vector. Data are mean ± SD from at least three independent cultures, each performed in triplicate. ***P < 0.001; **P < 0.01; *P < 0.05 (ANOVA plus post hoc). (D) Simultaneous deactivation of the TOR (by 100 nm torin) and Snf1/AMPK pathways leads to a strong downregulation of almost all metabolic activities. Color of the squares on the heat map corresponds to the mean value of the log fold change from three biological and three technical replicates. UBC6 was used for normalization. Control is wild‐type yeast with an empty vector. (E) The level of Snf1 phosphorylation upon simultaneous enrichment in each studied chaperone and the Hsp82 remains at the level observed in the control strain (Hsp82 enrichment in the wild‐type yeast background) and does not undergo an increase. Data represent quantification (ImageJ) of Western blot results from three independent experiments. Phospho‐Snf1 band intensity was normalized to the intensity of total Snf1 and β‐actin (loading control). Data are mean ± SD from at least three independent cultures, each performed in triplicate. ***P < 0.001; **P < 0.01; *P < 0.05 (ANOVA plus post hoc). (F) The amount of newly translated proteins upon simultaneous enrichment in each studied chaperone and the Hsp82 does not display the characteristic decrease, but rather a slight increase in translation activity. Data represent quantification (ImageJ) of Western blot results from three independent experiments (mean with SD). ***P < 0.001; **P < 0.01; *P < 0.05 (ANOVA plus post hoc). (G) Inhibition of Hsp82 by geldanamycin in the wild‐type strain (control) leads to the differential gene expression characteristic of ChES. Such gene expression pattern is consistent with TOR deactivation, albeit absent in the case of geldanamycin treatment of the strain with constitutively active TOR activity. Color of the squares on the heat map corresponds to the mean value of the log fold change from three biological and three technical replicates. UBC6 was used for normalization. (H) Inhibition of Hsp82 by geldanamycin leads to an increase in the oxygen consumption in the control strain, however, not in the strain with constitutively active TOR (caTor1). Control is wild‐type yeast. Data are mean ± SD from at least three independent cultures, each performed in triplicate. ***P < 0.001; **P < 0.01; *P < 0.05 (ANOVA plus post hoc).

To further elucidate the role of Tor1‐Snf1 cross talk in mitochondrial activity, we have measured oxygen consumption in ΔTor1 mutant, as well as in ΔSnf1 mutant treated with 100 nm torin, an inhibitor of TORC1. It should be noted that treating ΔSnf1 with torin is an experiment designed to replace the double deletion mutant ΔTor1ΔSnf1, which has proven difficult to make, presumably due fitness defects or no viability. Importantly, ΔTor1 mutant as well as the wild‐type yeast treated with torin displays consistent phenotype, as detailed below. The oxygen consumption in the ΔTor1 mutant as well as in the wild‐type strain treated with torin is increased by almost twofold, similar to the ChES strains (Fig. 5A). This increase in oxygen consumption is abrogated in the torin‐treated ΔSnf1. Moreover, the differential gene expression of the ΔTor1 mutant and the wild‐type strain treated with torin resembles the one of the ChESs: Increased expression levels of the respiratory chain complexes, as well as glycolysis repression, are the major features of this mutant (Figs 5B and S5G, Supporting information). Upon chaperone enrichment in the ΔTor1 background, no changes in the oxygen consumption or differential gene expression can be observed relative to the ones of the ΔTor1 strain (Fig. 5B,C). In the torin‐treated ΔSnf1, the differential gene expression displays a strong repression of glycolysis, TCA cycle, as well as respiration, consistent with the growth arrest and cell death observed in this condition. This strongly suggests that the TORC1 activity is essential for the viability/survival of the ΔSnf1 mutant (Figs 5D and S5H, Supporting information).

### Tor1 kinase activity responds to the level of Hsp82

Next, we set out to tackle the question of how TORC1 senses the protein folding environment. As downregulation of the Hsp82 level was observed in ChES, we hypothesized its role in relaying the information on protein folding status to TORC1. Therefore, we overexpressed Hsp82 (an average of twofold increase in expression level, Fig. S1A, Supporting information), in combination with each of the other chaperones, and measured the level of Snf1 activation via its phosphorylation. The results show that Snf1 phosphorylation increase, typical of the ChES, is absent when Hsp82 is overexpressed as well (Figs 5E and S6A, Supporting information). Further, the level of newly translated proteins is slightly increased upon overexpression of Hsp82 in the control strain; however, it remains unchanged when each of the studied chaperones is overexpressed together with Hsp82, suggesting that TORC1 deactivation is absent (Figs 5F and S6B). These results point toward the connection between Hsp82 downregulation and TORC1 deactivation in ChES and strongly suggest that Tor1 kinase senses the protein folding environment via the level of Hsp82.

Crucially, these results demonstrate that TORC1 acts as a ‘messenger’ between the folding environment and metabolism: Itself responsive of the status of folding environment, TORC1 is able to communicate with Snf1 kinase via its negative regulation.

To further verify the involvement of Hsp82 in the TORC1‐related chaperone activity sensing, we have used geldanamycin, an Hsp82 inhibitor, and measured the differential gene expression and mitochondrial activity under these conditions. We have found that Hsp82 inhibition by geldanamycin results in similar changes in gene expression as those observed in the ChES (Figs 5G and S6C, Supporting information), with glycolysis and TCA cycle repression and respiration activation being the main features. These results demonstrate that Hsp82 inhibition/downregulation may be one of the most proximal events of this signaling cascade. Moreover, this scenario is further corroborated by the increased oxygen consumption in the wild‐type strain treated with geldanamycin (Fig. 5H). Interestingly, fatty acid oxidation‐related *POT1* and *POX1* display a significant upregulation upon geldanamycin treatment, contrary to the previous results showing that FA oxidation increase is not related to enriched chaperone activity and reduced Hsp82 levels in the wild‐type background (ChES). On the other hand, when treated with geldanamycin, the strain exhibiting constitutive Tor1 activity (caTor1) does not experience the same changes: The differential gene expression is similar to the one of the caTor1 strain in the absence of geldanamycin and is characterized by upregulation of glycolytic enzymes and an overall unchanged expression levels of key components of the respiratory chain (Fig. 5G). Moreover, geldanamycin treatment of the caTor1 strain does not result in increased oxygen consumption relative to the caTor1 in the absence of geldanamycin and to the wild‐type control strain (Fig. 5H). These results further demonstrate that Hsp82 inhibition is a trigger event leading to TORC1 deactivation, Snf1 activation, and the consequent glucose starvation‐like phenotype in ChES. Furthermore, these results underscore the importance of TORC1 deactivation in the observed signaling cascade.

### Chaperone enrichment results in RLS extension

**Figure 6:**
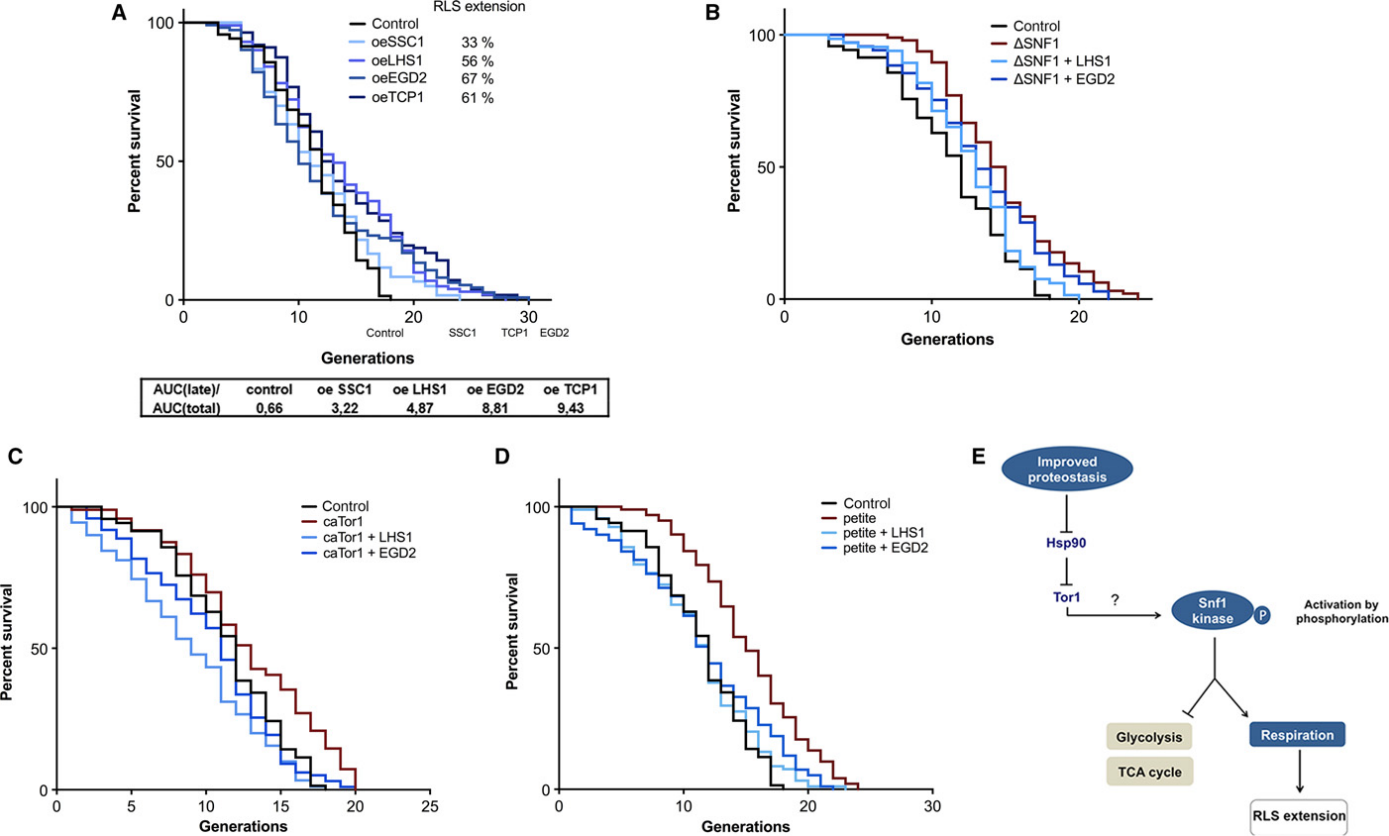
Replicative lifespan extension relies on TOR deactivation and Snf1 activation and is caused by the respiration increase in ChES. (A) Replicative lifespan is extended in ChES. The number of cells is 103, 120, 112, 129, and 127 for the wild‐type with an empty plasmid, oe SSC1, oe LHS1, oe EGD2, and oe TCP1, respectively. The table below the graph displays the percentage of the studied population that is still alive in the late life stage of the RLS. *P* values are 0.0009 (LHS1 and EGD2), 0.0004 (TCP1), and 0.0081 (0.0081). (B) Replicative lifespan extension in ChES is abolished in the absence of Snf1. The number of cells is 103 (control, wild‐type with an empty plasmid), 95 (ΔSnf1), 79 (ΔSnf1+LHS1), 95 (ΔSnf1+EGD2). *P* value for ΔSnf1 relative to control RLS is <0.0001. *P* values for ΔSnf1+LHS1 and ΔSnf1+EGD2 relative to the control are >0.05. (C) Replicative lifespan extension in ChES is abolished when the TOR pathway displays constitutive activity. The number of cells is 103 (control, wild‐type with an empty plasmid), 94 (caTor1), 89 (caTor1+LHS1), 94 (caTor1+EGD2). *P* values for the RLS of caTor1, caTor1+LHS1, and caTor1+EGD2 compared to the control are >0.05. (D) Replicative lifespan extension in ChES fully relies on the respiration increase and is absent in the case of chaperone enrichment in the respiration‐deficient background petite. The number of cells is 103 (control, wild‐type with an empty plasmid), 100 (petite), 97 (petite+LHS1), 99 (petite+EGD2). *P* value for the petite strain relative to control is <0.0001. *P* values for petite+LHS1 and petite+EGD2 relative to control are 0.048 and 0.044, respectively. The data shown are pooled from three independent experiments for each strain. Oe stands for overexpression. Significance of the results was tested with log‐rank (Mantel–Cox) test. (E) The proposed mechanism of enhanced chaperone activity sensing via Tor1‐Snf1 cross talk. Tor1 is in charge of sensing of improved protein folding environment via Hsp82, resulting in its decreased activity. The consequent activation of Snf1 kinase results in repression of glycolysis and the TCA cycle, as well as activation of respiration. Increased respiratory activity triggers replicative lifespan extension.

Replicative lifespan (RLS) is measured as the maximum number of divisions mother cells go through before the onset of senescence. The control strain produced a maximum of 18 buds during its RLS (Fig. 6A). We acknowledge that this RLS is shorter than reported in the literature for a related S288C wild‐type strain. However, one possible reason for this is the fact that we have monitored RLS constantly at 30 °C and the plates with cells were never placed to 4 °C during their lifespans, a widely used RLS extending practice.

The largest effect on RLS, with a 67% lifespan extension compared to the empty vector control, was achieved by overexpressing the Egd2, a subunit of the nascent polypeptide‐associated complex (NAC). Overexpression of Tcp1, cytosolic T‐complex, also had a beneficial effect on RLS, extending it by 61% compared to the control, as well as Lhs1, an ER Hsp70 chaperone, which yielded 56% RLS increase. Finally, the overexpression of Ssc1, the mitochondrial HSP70, resulted in 33% lifespan extension relative to the empty vector control. Median RLS is not affected and is ~12 generations for the studied strains (Fig. 6A).

The observed extension of the RLS in ChES is mainly limited to the final part of the survival curve. Therefore, to exclude the possibility that the effect we see is a consequence of several resistant cells refusing to die, normally present in each population, we have calculated the area under the curve corresponding to the final lifespan stage (AUC_late_) (see Methods for details). The AUC_late_, normalized to the total AUC under the entire survival curve (AUC_total_), in the control strain reports that 0.66% of the studied population reaches the late life stage. On the other hand, ChESs show an increase in this parameter, with 4–10% of the population reaching the late life stage in the four ChESs. This result further speaks in favor of the increased longevity of the ChES, while the question remains what is its underlying mechanism.

To facilitate the comparison with the literature, we have performed a subset of RLS measurements at 30 °C with overnight storing of plates at 4 °C. Our control strain (wild‐type with the empty vector) displays a mean RLS of ~30 generations with a maximum RLS of ~50 generations (Fig. S7A, Supporting information). Compared to the same strain with continuous monitoring at 30 °C, this represents a dramatic extension of RLS. Enrichment in Egd2 and Lhs1 further extends the RLS with mean RLS around 40 generation and maximum RLS more than 60 generation (Fig. S7A, Supporting information). As these results clearly demonstrate that overnight refrigeration of plates yields an effect on yeast RLS, which has yet to be investigated, we continue to present the RLS measurements with constant monitoring at 30 °C. Such effect of overnight refrigeration is not surprising: It is well known that extended periods (4–12 h) at 4 and 10°C in budding yeast induce the expression of many chaperones (Hsp26, Hsp42, Hsp104, Hsp70), trehalose synthesis genes, and antioxidant protection from which the cell can and does benefit immensely (Aguilera *et al*., 2007).

### Snf1/AMPK activation is essential for the chaperone‐mediated lifespan extension

To test its involvement in the RLS extension, we determined RLS of ChES in the absence of Snf1 kinase. As all the strains tested so far displayed similar phenotype, we decided to limit the laborious RLS measurements to ChES overexpressing Lhs1 and Egd2. The deletion of Snf1 kinase itself resulted in an extended RLS with the maximum number of generations of 24 and the median of 15 generations. Although this is not a large RLS extension, the displayed trend is opposite from the published data that report on a decreased RLS in the absence of Snf1 (Ashrafi *et al*., 2000). One potential reason could be the difference in experimental conditions of micromanipulation that, in our case, was performed continuously at 30 °C, and not with overnight refrigeration as in Ashrafi *et al*. However, in the absence of Snf1, RLS of these two ChES mutants is comparable to that of the control strain; that is, there is a loss of the chaperone‐mediated RLS extension when compared to the ChES (Fig. 6B, compared to Fig. 6A). Thus, we conclude that the Snf1 kinase is essential for the observed RLS extension.

### Tor1 deactivation is essential for the chaperone‐mediated lifespan extension

To investigate the role that TOR deactivation has in the RLS extension observed in ChES, we have overexpressed each chaperone in the background with constitutively active Tor1 kinase (caTor1) background. As above, we have limited the RLS measurement to the strains enriched in Lhs1 and Egd2.

The maximum number of generations that the caTor1 strain goes through is 20, thereby displaying no significant difference from the control strain (Fig. 6C). Moreover, enrichment in Lhs1 and Egd2 in the caTor1 background did not yield any changes in maximum RLS compared to the control, however, and led to a strong reduction of RLS compared to the one observed in ChES. The observed maximum number of generations is 18 for Lhs1 enrichment and 20 for Egd2 enrichment in the background of caTor1 (Fig. 6C). In the case of Lhs1 enrichment, the median RLS has decreased to only nine generations. Based on these results, we conclude that the deactivation of Tor1 kinase is an essential part of the mechanism responsible for the observed RLS extension.

### Respiration increase is essential for the chaperone‐mediated lifespan extension

Respiration increase during carbon starvation (CS) has been identified as the most likely event responsible for the CS‐driven RLS extension (Lin *et al*., 2002). To investigate whether that is also the case in ChES, we have overexpressed the chaperones in the respiration‐deficient, petite (ρ‐) strain. While the petite strain itself displays an extended median and maximum RLS relative to the control strain (16 and 24 generations, respectively), consistent with previous observations (Woo & Poyton, 2009), the chaperone enrichment in the petite background does not result in further RLS extension, with only 23 (Lhs1 enrichment) and 24 (Egd2 enrichment) generations (Fig. 6D). This result underscores the importance of respiration increase during chaperone enrichment in the RLS extension.

## Discussion

The relationship between cellular proteostasis and the metabolic processes can be considered a central homeostatic mechanism, and its importance is unequivocal. Yet, almost nothing is known about this connection and its role in cellular functioning. More specifically, it is vague which pathway performs the sensing of the folding environment, as well as if and how such information is relayed to other cellular networks. Furthermore, while it is rather well described how a cell responds to proteotoxic stress in the context of stress responses, nothing is known about cellular response to alleviation of protein misfolding.

Here, we present a new perspective from which cellular proteostasis is implicated in basic cellular functioning via its cross talk with metabolism and mitochondrial function. We demonstrate that protein chaperones are not only managers of misfolded proteins, but also act as some of the key determinants of cellular metabolic activity. Although we cannot rule out the possibility that the four chaperones employed in this study have specific regulatory roles regarding Hsp82, our results suggest that alleviation of protein misfolding impacts mitochondrial function and metabolic activity.

The transcriptome analysis reveals that the chaperone‐enriched strains (ChESs) have activated a response that is a phenocopy of the response to glucose starvation and that relies on their chaperoning activity. This response is characterized by a reduction in the glycolytic and the TCA cycle activity accompanied by an increase in oxygen consumption (Lin *et al*., 2002).

Our results implicate Snf1 kinase in the integration of signals of protein folding environment status with carbon metabolism (Fig. 6E). As evidenced by the differential gene expression characteristic of chaperone enrichment in the absence of Snf1, Snf1 activation is responsible for the metabolic reprogramming observed in ChES. Usually, cell growth in limited glucose availability activates the Snf1 kinase by its phosphorylation (Hong *et al*., 2003), resulting in transcriptional activation of genes encoding for enzymes involved in gluconeogenesis, fatty acid oxidation, and respiration (Busti *et al*., 2010), as well as repression of glycolysis. A response reciprocal to this one was observed in our previous work: Mild proteotoxic stress resulted in a decline in respiration, activation of the pentose phosphate pathway as well as induction of antioxidative protection and protein maintenance machineries (Perić *et al*., 2016).

Furthermore, our results indicate that when the folding environment is impeccable, the cell reduces the level of new protein synthesis. Presumably, there are two possible reasons for this: First, the energy is invested into protein maintenance rather than synthesis, and second, the damage‐induced protein degradation may be decreased, also resulting in the lower need for new synthesis. In this context, we uncovered a dual role of the TORC1 complex (Fig. 6E):

1. Tor1 kinase as a messenger delivering the information on the status of the folding environment as the negative regulator of Snf1 kinase.
2. Tor1 kinase as a sensor of the folding environment improvement, resulting in its deactivation.

The interplay between the TOR pathway and proteostasis has already been studied in human cultured cells where a key role of the mTORC1 has been proposed in response to proteotoxic stress (Chou *et al*., 2012). Further, it has been demonstrated that reduction in mTORC1 levels by RNA interference leads to increased sensitivity to heat shock. This effect was accompanied by a drastic reduction in ability to synthesize heat‐shock proteins (HSPs), including Hsp70, Hsp90, and Hsp110 (Chou *et al*., 2012). Another study demonstrated that cells are able to distinguish between moderate and severe proteotoxicity and that mTORC1 complex is in charge of sensing the misfolded proteins (Qian *et al*., 2010).

Levels of TOR pathway activity directly modulate cellular metabolism, in part by shifting the balance between modes of energy production and usage. We have shown that chaperone gain of function indeed decreases TOR activity, thereby decreasing the amount of newly synthesized proteins, and, on the other hand, activating Snf1 kinase and a glucose starvation‐like response. Consistent with our observations, previously obtained immunochemical data in human cell cultures indicate that nitrogen limitation improves Snf1 phosphorylation, thereby showing that Snf1 is negatively regulated by the rapamycin‐sensitive Tor kinase (Orlova *et al*., 2006). As TORC1 deactivation and Snf1 activation are responses to nitrogen and carbon starvation, respectively, the possibility that TORC1 deactivation may serve as a signal for Snf1 activation is rather expected, albeit different from their canonical relationship that posits Snf1 upstream of TOR. Moreover, it has been shown that a decreased activity of Tor1 in yeast results in a greater activity of the mitochondrial respiratory chain, observed as an increased oxygen consumption, as well as ROS level (Pan *et al*., 2011). The authors propose that the observed increase in chronological lifespan may be a consequence of an adaptive response of cells to the increased ROS level. However, based on our results, it is possible that the increased lifespan in the Tor1‐deficient strain is a result of Snf1 activation leading to a response akin to glucose starvation. Interestingly, our results suggest that simultaneous inactivation of both TORC1 and Snf1 pathways leads to severe growth defects and cell death. This result suggests that cells cannot tolerate simultaneous repression of both glycolysis (consequence of TORC1 deactivation) and autophagy (consequence of Snf1 deletion). Details of this cross talk will be a subject of extensive research in the future.

Dynamic nature of the TORC1 complex, whose assembly is necessary for signal transduction, could be responsible for the plasticity of communication between proteostasis and metabolism (Sengupta *et al*., 2010). In the context of protein folding sensing, we identify the Hsp82 chaperone as a TOR informant on the folding status, consistently with multiple functions that this chaperone has been proposed to have in the context of cellular metabolism (Dezwaan & Freeman, 2008). Simultaneous enrichment of cells with Hsp82 together with each studied chaperone does not result in the response akin to glucose starvation, suggesting that the decline in Hsp82 activity is required to inactivate TORC1 and trigger the downstream phenotype. Inhibition of Hsp82 by geldanamycin confirms such scenario: Upon chemical inactivation of Hsp82 in the wild‐type yeast, a response consistent with one in ChES is observed. The differential gene expression reveals repression of glycolysis and increase in the expression of the respiratory chain complexes, supported by increased oxygen consumption. Importantly, such effect of Hsp82 inhibition by geldanamycin is absent in the strain with constitutive Tor1 kinase activity, demonstrating that the inactivation of TORC1 is essential for relaying the information on Hsp82 deactivation. mTOR and other regulatory components, such as raptor, are chaperone clients in mammalian cells, as evidenced by their selective binding to Hsp90 when not forming a complex (Qian *et al*., 2010). Such mechanism likely enables yeast TORC1 to rapidly detect and respond to intracellular folding environment, while also adjusting the cellular metabolic activity. The tight linkage between protein quality and quantity control provides a plausible mechanism coupling protein folding quality with metabolic activity.

Many conditions that shift cells from states of nutrient utilization and growth to states of cell maintenance extend lifespan across species (Lakowski & Hekimi, 1998; Kaeberlein *et al*., 2006; Grandison *et al*., 2009). In yeast, the most common way to invoke dietary restriction involves reduced glucose or amino acid levels in the medium (Lin *et al*., 2000; Fabrizio *et al*., 2004). In other words, improved proteostasis, TOR deactivation (typical for amino acid starvation), and Snf1 activation (typical for glucose starvation response) all could have been responsible for the observed RLS extension in ChES. We show that the observed RLS extension relies completely on enhanced respiratory activity of the ChES, one of the metabolic consequences of improved proteostasis. In the proposed mechanism (Fig. 6E), enhanced respiration is a downstream consequence of TORC1 deactivation and Snf1 activation, evidenced by the loss of the chaperone‐mediated RLS extension in the absence of Snf1 and in the background of constitutively active Tor1 kinase. The involvement of Snf1 in aging has already been described with its deletion and overexpression shortening and extending the lifespan, respectively (Ashrafi *et al*., 2000; Managbanag *et al*., 2008). Similarly, it has been shown that the deletion of Snf1 in budding yeast abolishes the proteasome‐mediated lifespan extension (Yao *et al*., 2015).

The results presented here shift the focus away from the well‐known role of chaperones in protein homeostasis, while highlighting their downstream effects on cellular metabolic and mitochondrial activity. Specifically, a persistent theme in the studies of aging is the contribution of energy intake and expenditure to longevity on one hand, and protein homeostasis on the other. Our study suggests that these two pathways are not only separately involved in aging, but that there is a cross talk between them, promoted by the communication between Tor1 and Snf1 kinases, resulting in the activation of the latter regardless of glucose availability. We suggest that protein chaperones should be included among the molecules that are able to modulate glucose sensing and hence the energy status of the cell. Although the purpose of activating an energy‐saving response as a consequence of protein misfolding alleviation remains elusive, the described mechanism may help direct the search for caloric restriction mimetics and provide novel avenues for future research of aging and age‐related pathologies.

## Experimental procedures

### Strains and growth conditions

Wild‐type *Saccharomyces cerevisiae* Y258 was used, derived from S288C. WT Y258 and strains overexpressing Lhs1, Ssc1, Egd2, and Tcp1 genes were purchased from Thermo Scientific (Dharmacon, Lafayette, Colorado, USA). The overexpression of each chaperone was confirmed using the RNASeq data as well as by flow cytometry (details below). WT Y258 was grown on YPD medium with 2% (w/v) glucose and chaperone overexpression mutants on –URA medium with 2% (w/v) glucose, at 30 °C with shaking. Hsp82 (an Hsp90 chaperone, YPL240C) overexpression was applied from the same plasmid in which the *URA3* was replaced by a nourseothricin cassette. The strain with constitutively active Tor1 kinase was selected on 9 mm caffeine YPD plates and confirmed by sequencing of the Tor1 locus to have the I1954V mutation. The sequence of Tor2 locus was confirmed to be unchanged. Deletion of Snf1 was performed according to the previously described protocol, using a hygromycin cassette for selection (Rothstein, 1983). Expression from plasmids was induced by 2% galactose using the following protocol: Overnight cultures were diluted 100× and grown until mid‐exponential phase. Then, the cells were pelleted by a 5‐min centrifugation at 4000 *g* and resuspended in YPD medium without glucose, supplemented with 2% galactose. The empty vector control is always treated in the same manner. All experiments were performed on postdiauxic shift cells, harvested by 5‐min centrifugation at 4000 *g*, washed, and treated accordingly. In different experiments, the identity of the control strain is defined in the Results section and in the corresponding figure caption.

All other methods are described in Appendix S1 (Supporting information).

## Funding

This work was supported by the Fondation Nelia et Amedeo Barletta, NAOS Group, and the Mediterranean Institute for Life Sciences to AV, MP, MM, MR, AN, and AK; Croatian Ministry of Science, Education and Sports, Grant No. 098‐0982464‐1647 to AŠ and SS; European Commission Seventh Framework Program, Integra‐Life; grant 315997 to KV; FP7‐REGPOT‐2012‐2013‐1, Grant No. 316289, InnoMol to AMM; grant 337327 from the European Research Council to NR and PBD; IM is supported by an Emmy Noether Award from the Deutsche Forschungsgemeinschaft.

## Author contributions

The study was designed by NR and AK; data were generated and analyzed by MP, AL, AŠ, SS, MR, MM, AN, PBD, AMM, SD, NR, and AK; consultation on spinning disk imaging was provided by IM; RNA sequencing data analysis was designed and conducted by KV; the manuscript was written by NR, KV, and AK.

## Conflict of interest

The authors have no conflict of interest to declare.

## Supporting information


**Fig. S1** Enrichment in four different chaperones from different cellular compartments results in alleviation of protein stress.
**Fig. S2** Examples of representative images of Hsp104‐GFP tagged protein aggregates.
**Fig. S3** The schematic presentation of domain architecture of each studied chaperone.
**Fig. S4** Chaperone activity is critical for the induction of the glucose starvation‐like response.
**Fig. S5** TOR deactivation and consequent Snf1 activation are key events leading to the glucose starvation‐like response in ChES.
**Fig. S6** Hsp82 activity reduction results in Tor1 deactivation.
**Fig. S7** Replicative lifespan of ChES with overnight storage of plates at 4 °C. Principle component analysis‐based filtering of RNASeq replicates.

**Table S1** The summary of the RNA Sequencing results with the data for each strain organized in separate tabs.

**Table S2** The list of genes with their transcript levels measured by qPCR in this study and used primers.

**Appendix S1** Experimental procedures.

## References

[acel12623-bib-0001] Aguilera J , Randez‐Gil F , Prieto JA (2007) Cold response in *Saccharomyces cerevisiae*: new functions for old mechanisms. FEMS Microbiol. Rev. 31, 327–341. 17298585 10.1111/j.1574-6976.2007.00066.x

[acel12623-bib-0002] Andersson V , Hanzen S , Liu B , Molin M , Nystrom T (2013) Enhancing protein disaggregation restores proteasome activity in aged cells. Aging 5, 802–812. 24243762 10.18632/aging.100613 PMC3868723

[acel12623-bib-0003] Ashrafi K , Lin SS , Manchester JK , Gordon JI (2000) Sip2p and its partner Snf1p kinase affect aging in *S. cerevisiae* . Genes Dev. 14, 1872–1885. 10921902 PMC316829

[acel12623-bib-0004] Brandvold KR , Morimoto RI (2015) The chemical biology of molecular chaperones–implications for modulation of proteostasis. J. Mol. Biol. 427, 2931–2947. 26003923 10.1016/j.jmb.2015.05.010 PMC4569545

[acel12623-bib-0005] Busti S , Coccetti P , Alberghina L , Vanoni M (2010) Glucose signaling‐mediated coordination of cell growth and cell cycle in *Saccharomyces cerevisiae* . Sensors 10, 6195–6240. 22219709 10.3390/s100606195 PMC3247754

[acel12623-bib-0006] Chou SD , Prince T , Gong J , Calderwood SK (2012) mTOR is essential for the proteotoxic stress response, HSF1 activation and heat shock protein synthesis. PLoS ONE 7, e39679. 22768106 10.1371/journal.pone.0039679 PMC3387249

[acel12623-bib-0007] Dezwaan DC , Freeman BC (2008) HSP90: the Rosetta stone for cellular protein dynamics? Cell Cycle 7, 1006–1012. 18414022 10.4161/cc.7.8.5723

[acel12623-bib-0008] Fabrizio P , Battistella L , Vardavas R , Gattazzo C , Liou LL , Diaspro A , Dossen JW , Graua EB , Longo VD (2004) Superoxide is a mediator of an altruistic aging program in *Saccharomyces cerevisiae* . J. Cell Biol. 166, 1055–1067. 15452146 10.1083/jcb.200404002 PMC2172019

[acel12623-bib-0009] Grandison RC , Piper MD , Partridge L (2009) Amino‐acid imbalance explains extension of lifespan by dietary restriction in *Drosophila* . Nature 462, 1061–1064. 19956092 10.1038/nature08619 PMC2798000

[acel12623-bib-0010] Hardt M , Chantaravisoot N , Tamanoi F (2011) Activating mutations of TOR (target of rapamycin). Genes Cells 16, 141–151. 21210909 10.1111/j.1365-2443.2010.01482.x PMC3116645

[acel12623-bib-0011] Hedbacker K , Carlson M (2009) SNF1/AMPK pathways in yeast. Front Biosci. 13, 2408–2420. 10.2741/2854 PMC2685184 17981722

[acel12623-bib-0012] Hong SP , Leiper FC , Woods A , Carling D , Carlson M (2003) Activation of yeast Snf1 and mammalian AMP‐activated protein kinase by upstream kinases. Proc. Natl Acad. Sci. USA 100, 8839–8843. 12847291 10.1073/pnas.1533136100 PMC166400

[acel12623-bib-0013] Kaeberlein TL , Smith ED , Tsuchiya M , Welton KL , Thomas JH , Fields S , Kennedy BK , Kaeberlein M (2006) Lifespan extension in *Caenorhabditis elegans* by complete removal of food. Aging Cell 5, 487–494. 17081160 10.1111/j.1474-9726.2006.00238.x

[acel12623-bib-0014] Labbadia J , Morimoto RI (2015) The biology of proteostasis in aging and disease. Annu. Rev. Biochem. 84, 435–464. 25784053 10.1146/annurev-biochem-060614-033955 PMC4539002

[acel12623-bib-0015] Lakowski B , Hekimi S (1998) The genetics of caloric restriction in *Caenorhabditis elegans* . Proc. Natl Acad. Sci. USA 95, 13091–13096. 9789046 10.1073/pnas.95.22.13091 PMC23719

[acel12623-bib-0017] Lin SJ , Defossez PA , Guarente L (2000) Requirement of NAD and SIR2 for life‐span extension by calorie restriction in *Saccharomyces cerevisiae* . Science 289, 2126–2128. 11000115 10.1126/science.289.5487.2126

[acel12623-bib-0018] Lin SJ , Kaeberlein M , Andalis AA , Sturtz LA , Defossez PA , Culotta VC , Fink GR , Guarente L (2002) Calorie restriction extends *Saccharomyces cerevisiae* lifespan by increasing respiration. Nature 418, 344–348. 12124627 10.1038/nature00829

[acel12623-bib-0019] Managbanag JR , Witten TM , Bonchev D , Fox LA , Tsuchiya M , Kennedy BK , Kaeberlein M (2008) Shortest‐path network analysis is a useful approach toward identifying genetic determinants of longevity. PLoS ONE 3, e3802. 19030232 10.1371/journal.pone.0003802 PMC2583956

[acel12623-bib-0020] Morano KA , Liut PCC , Thiele DJ (1998) Protein chaperones and the heat shock response in *Saccharomyces cerevisiae* . Curr. Opin. Microbiol. 1, 197–203. 10066474 10.1016/s1369-5274(98)80011-8

[acel12623-bib-0021] Orlova M , Kanter E , Krakovich D , Kuchin S (2006) Nitrogen availability and TOR regulate the Snf1 protein kinase in *Saccharomyces cerevisiae* . Eukaryot. Cell 5, 1831–1837. 16980405 10.1128/EC.00110-06 PMC1694804

[acel12623-bib-0022] Pan Y , Schroeder EA , Ocampo A , Barrientos A , Shadel GS (2011) Regulation of yeast chronological life span by TORC1 via adaptive mitochondrial ROS signaling. Cell Metab. 13, 668–678. 21641548 10.1016/j.cmet.2011.03.018 PMC3110654

[acel12623-bib-0023] Perić M , Dib PB , Dennerlein S , Musa M , Rudan M , Lovrić A , Nikolić A , Šarić A , Sobočanec S , Mačak Ž , Raimundo N , Kriško A (2016) Crosstalk between cellular compartments protects against proteotoxicity and extends lifespan. Sci. Rep. 6, 28751. 27346163 10.1038/srep28751 PMC4921836

[acel12623-bib-0024] Qian SB , Zhang X , Sun J , Bennink JR , Yewdell JW , Patterson C (2010) mTORC1 links protein quality and quantity control by sensing chaperone availability. J. Biol. Chem. 285, 27385–27395. 20605781 10.1074/jbc.M110.120295 PMC2930736

[acel12623-bib-0025] Rothstein RJ (1983) One‐step gene disruption in yeast. Methods Enzymol. 101, 202–211. 6310324 10.1016/0076-6879(83)01015-0

[acel12623-bib-0026] Rowland MA , Fontana W , Deeds EJ (2012) Crosstalk and competitions in signalling network. Biophys. J. 103, 2389–2398. 23283238 10.1016/j.bpj.2012.10.006 PMC3514525

[acel12623-bib-0027] Sengupta S , Peterson TR , Sabatini DM (2010) Regulation of the mTOR complex 1 pathway by nutrients, growth factors and stress. Mol. Cell 40, 310–322. 20965424 10.1016/j.molcel.2010.09.026 PMC2993060

[acel12623-bib-0028] Su KH , Cao J , Tang Z , Dai S , He Y , Sampson SB , Benjamin IJ , Dai C (2016) HSF1 critically attunes proteotoxic stress sensing by mTORC1 to combat stress and promote growth. Nat. Cell Biol. 18, 527–539. 27043084 10.1038/ncb3335 PMC5341796

[acel12623-bib-0029] Vidovic A , Supek F , Nikolic A , Krisko A (2014) Signatures of conformational stability and oxidation resistance in proteomes of pathogenic bacteria. Cell Rep. 7, 1393–1400. 24882003 10.1016/j.celrep.2014.04.057

[acel12623-bib-0030] Wolff S , Weissman JS , Dillin A (2014) Differential scales of protein quality control. Cell 157, 52–64. 24679526 10.1016/j.cell.2014.03.007

[acel12623-bib-0031] Woo DK , Poyton RO (2009) The absence of a mitochondrial genome in rho0 yeast cells extends lifespan independently of retrograde regulation. Exp. Gerontol. 44, 390–397. 19285548 10.1016/j.exger.2009.03.001 PMC3341797

[acel12623-bib-0032] Yao Y , Tsuchiyama S , Yang C , Bulteau AL , He C , Robison B , Tsuchiya M , Miller D , Briones V , Tar K , Potrero A , Friguet B , Kennedy BK , Schmidt M (2015) Proteasomes, Sir2, and Hxk2 form an interconnected aging network that impinges on the AMPK/Snf1‐regulated transcriptional repressor Mig1. PLoS Genet. 11, e1004968. 25629410 10.1371/journal.pgen.1004968 PMC4309596

