# Supplementary figures and images for: TORC1‐mediated sensing of chaperone activity alters glucose metabolism and extends lifespan
Source: Aging Cell. 2017 Jun 14;16(5):994–1005. doi: 10.1111/acel.12623 (PMC5595670; doi:10.1111/acel.12623)

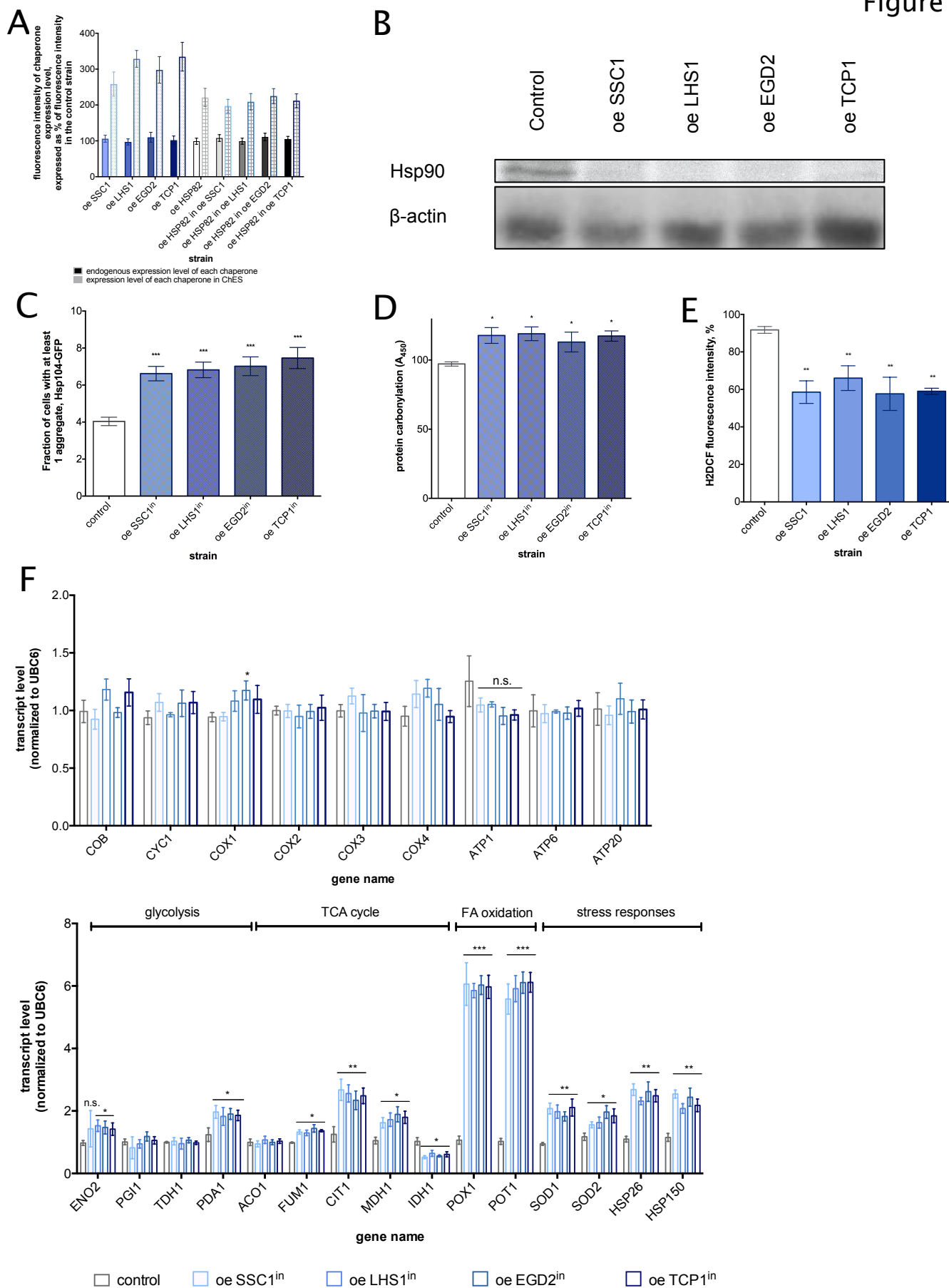

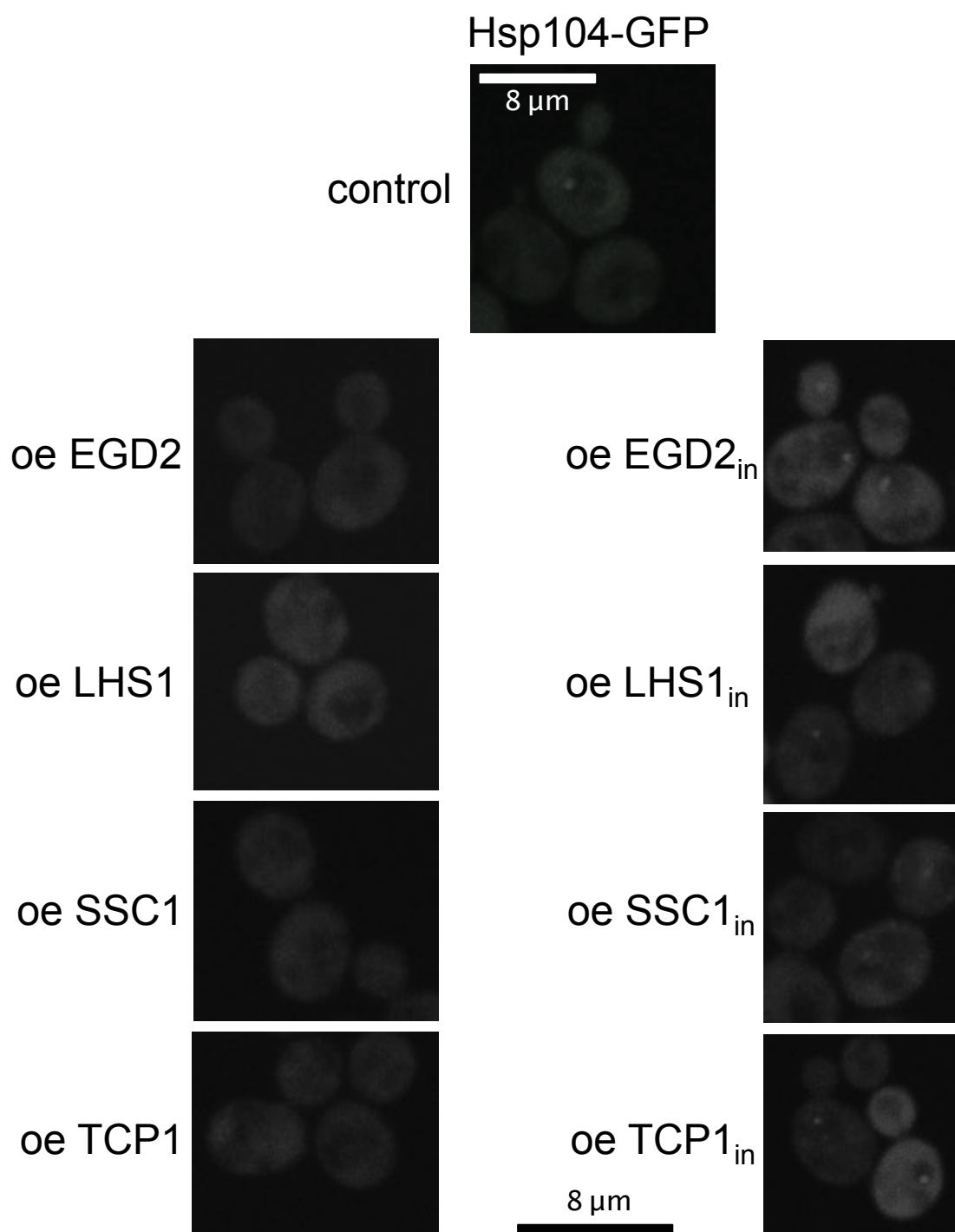

## SSC1

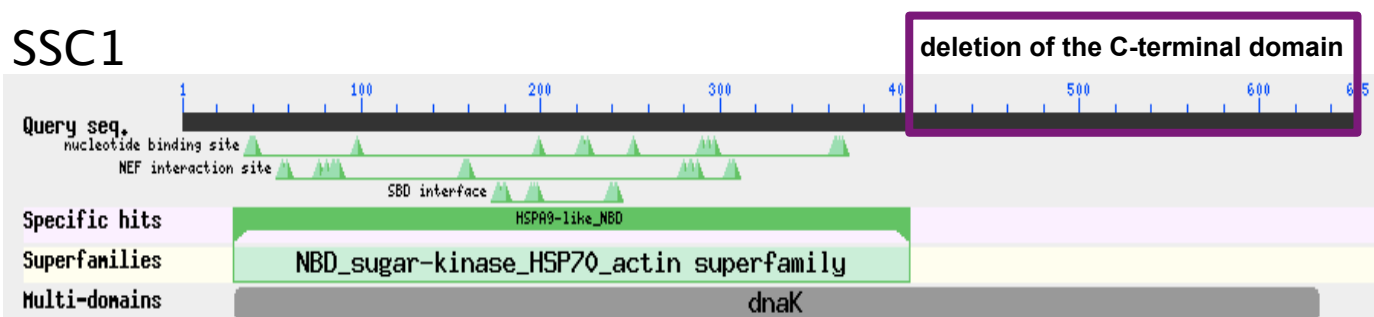

## LHS1

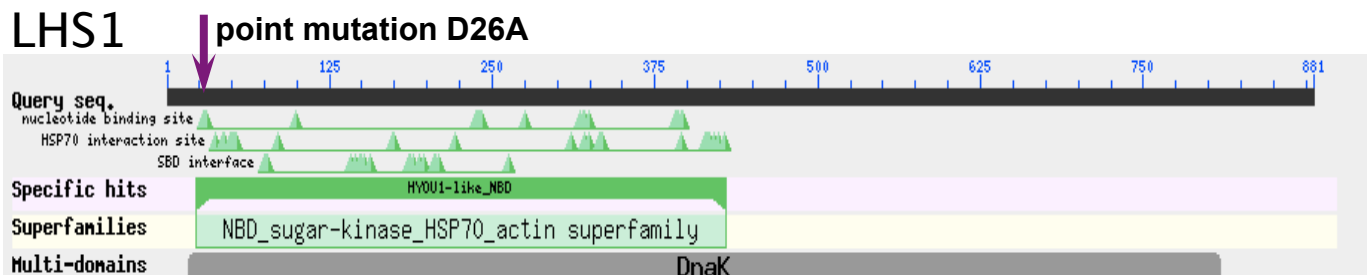

## EGD2

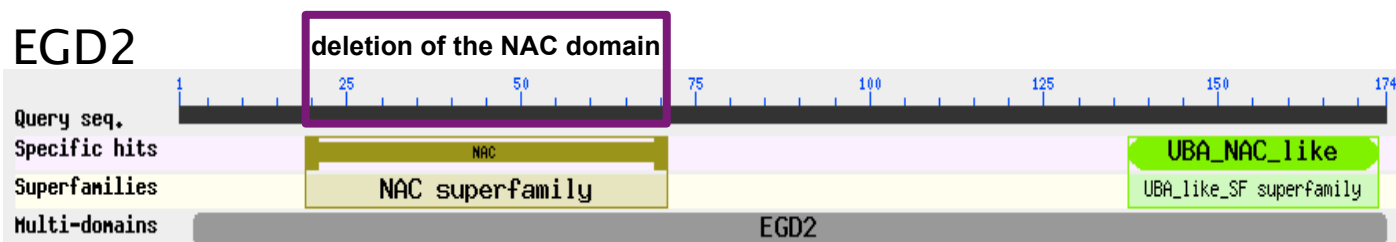

## TCP1

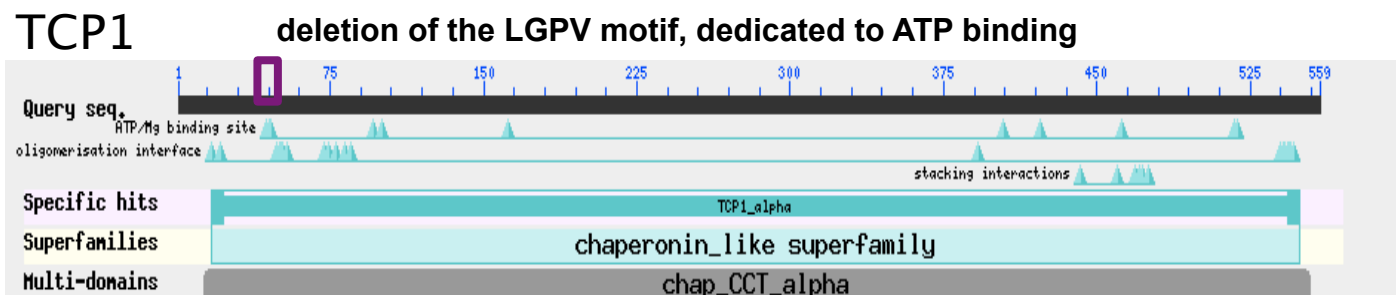

Figure S4

A

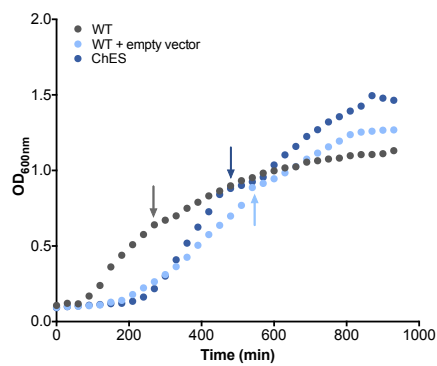

B

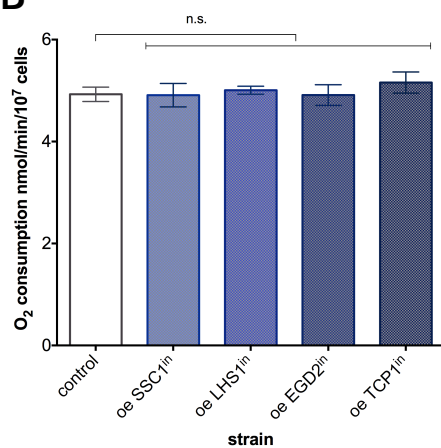

C

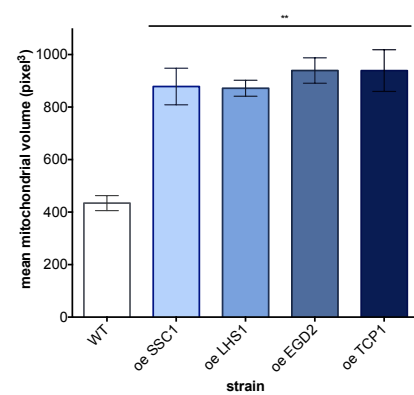

D

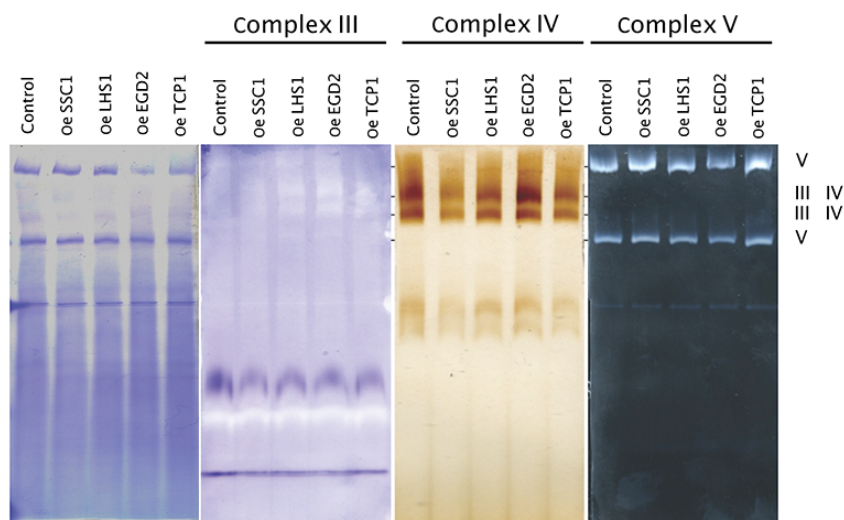

**A**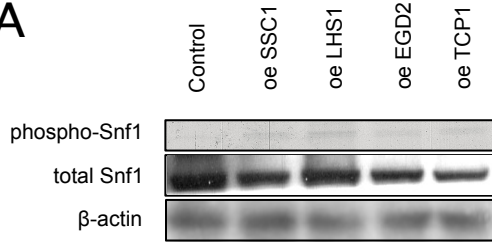**B**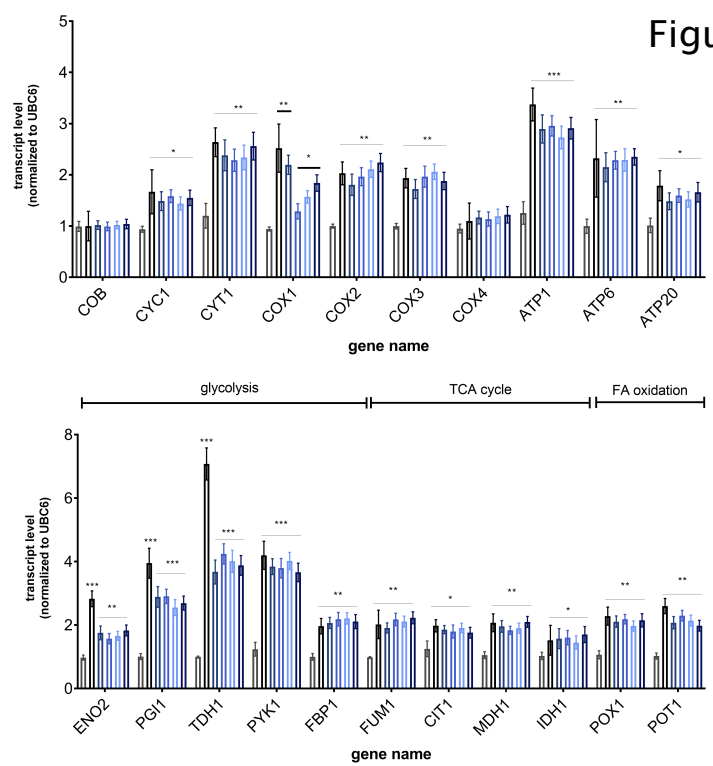**C**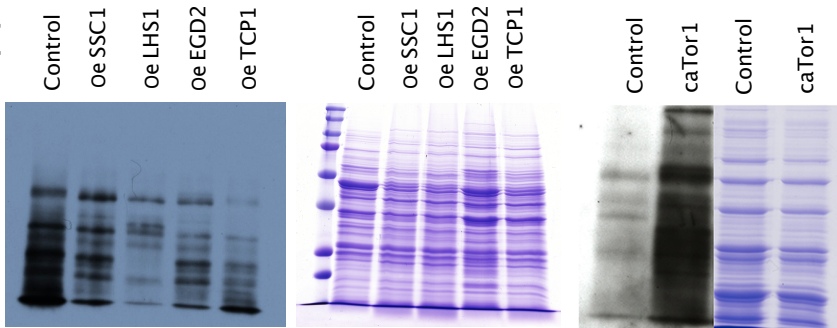**D**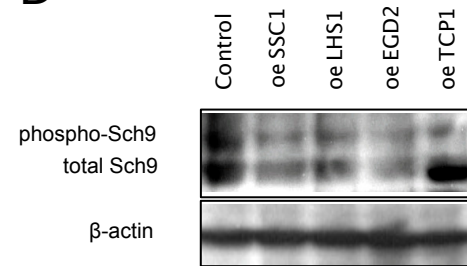**E**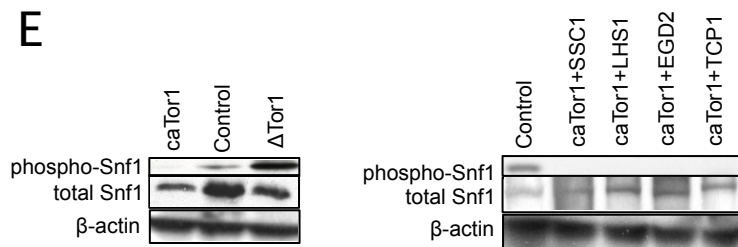**F**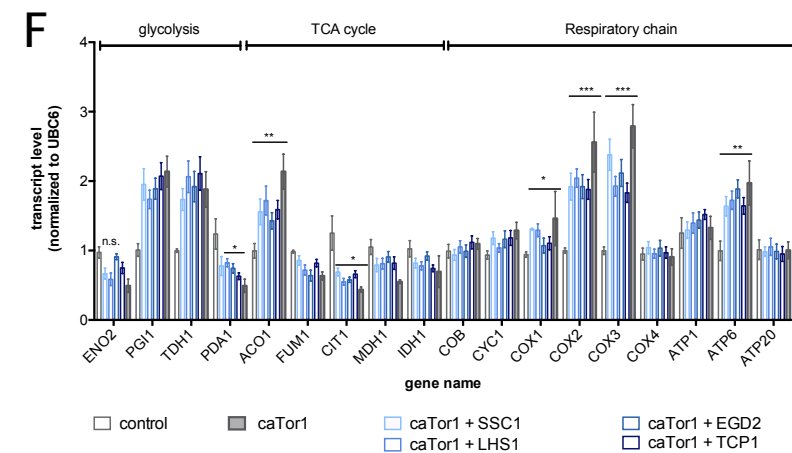

G

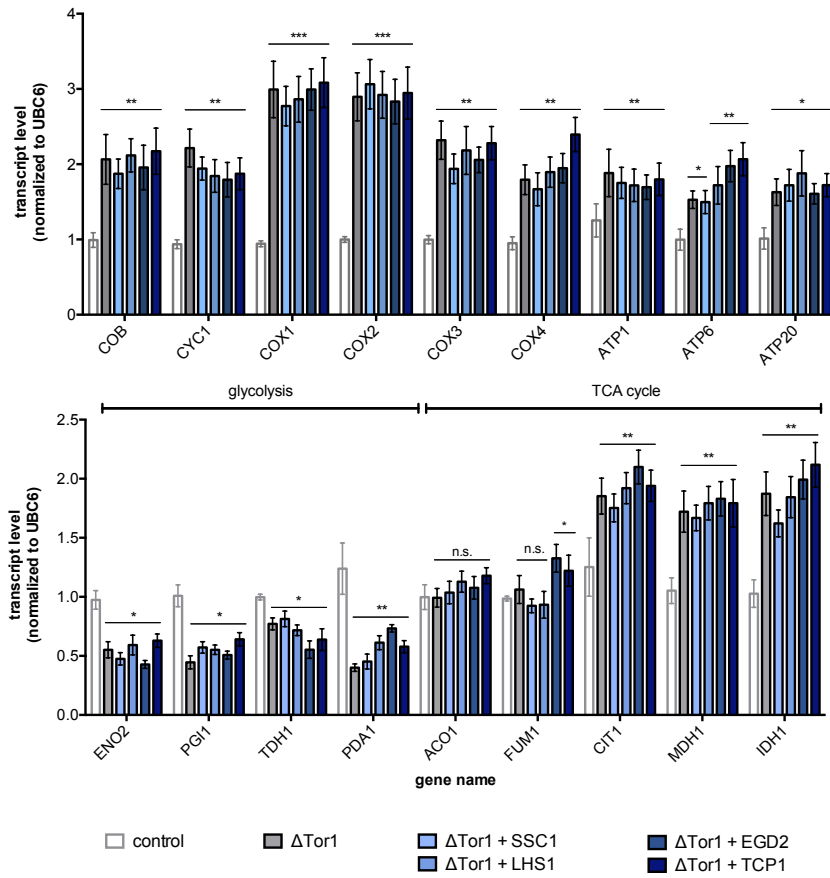

H

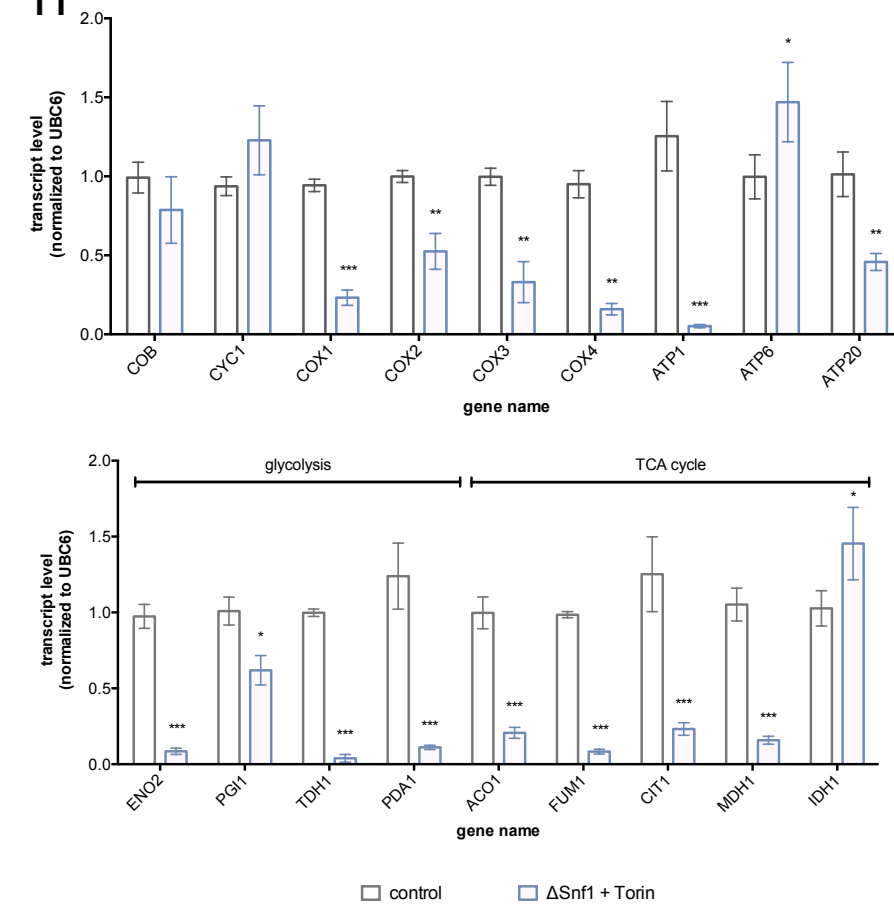

Figure S6

A

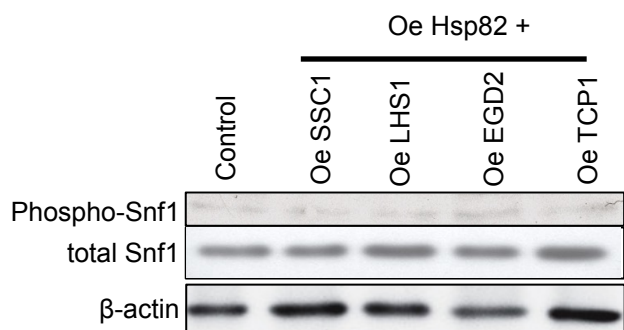

B

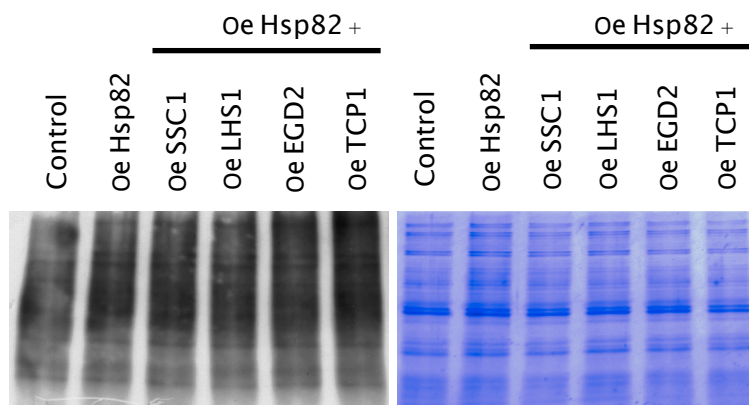

C

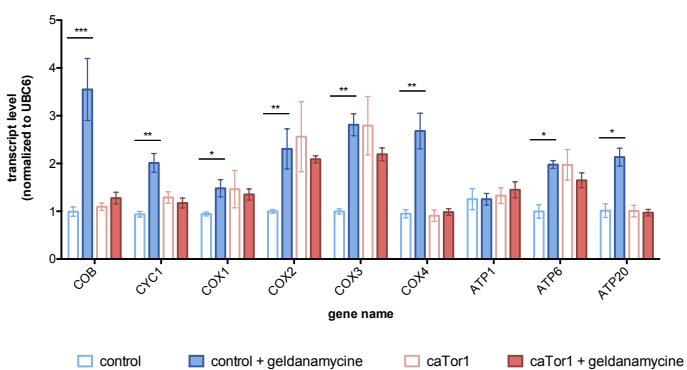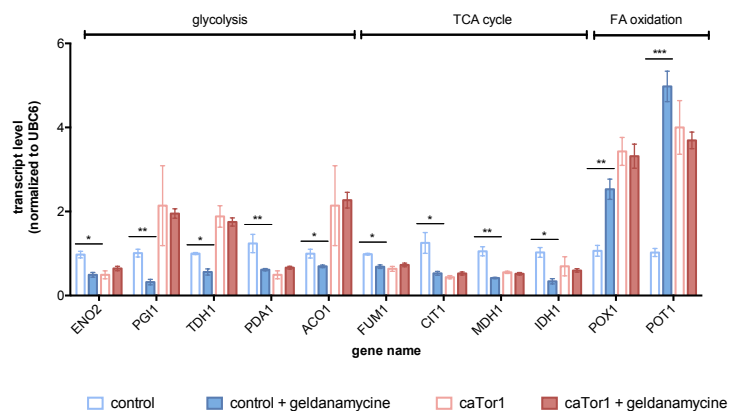

Figure S7

A

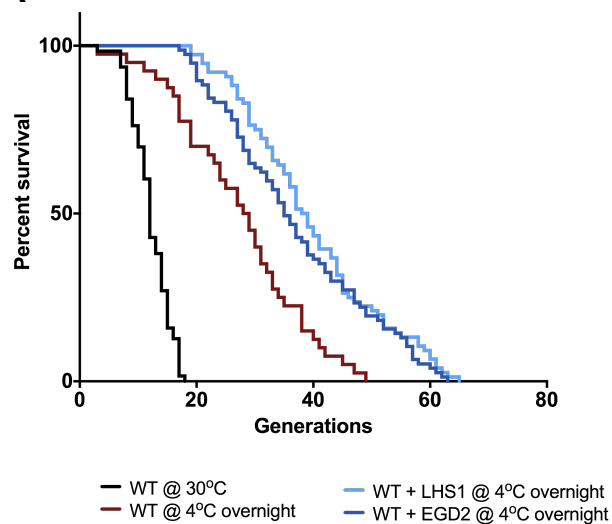

B

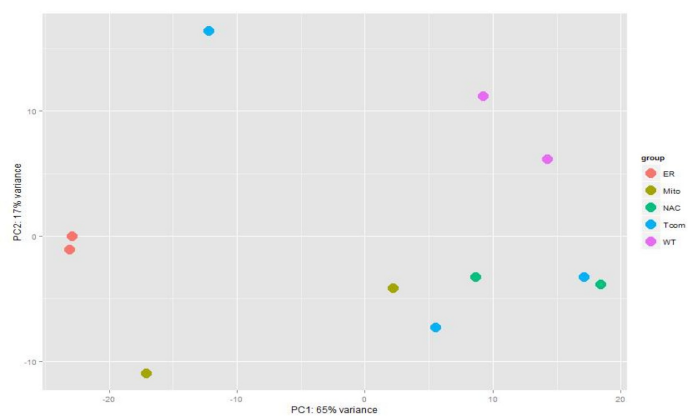

C

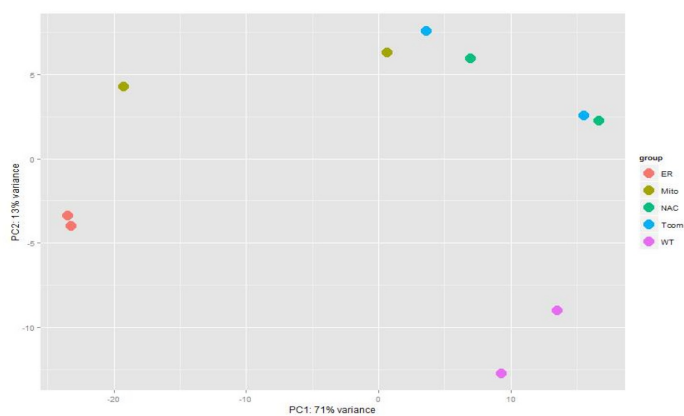

Supplement: Supplementary file 1 — Fig. S1 Enrichment in four different chaperones from different cellular compartments results in alleviation of protein stress. Fig. S2 Examples of representative images of Hsp104‐GFP tagged protein aggregates. Fig. S3 The schematic presentation of domain architecture of each studied chaperone. Fig. S4 Chaperone activity is critical for the induction of the glucose starvation‐like response. Fig. S5 TOR deactivation and consequent Snf1 activation are key events leading to the glucose starvation‐like response in ChES. Fig. S6 Hsp82 activity reduction results in Tor1 deactivation. Fig. S7 Replicative lifespan of ChES with overnight storage of plates at 4 °C. Principle component analysis‐based filtering of RNASeq replicates. [file ACEL-16-994-s001.pdf]
