# Supplementary material for: TORC1‐mediated sensing of chaperone activity alters glucose metabolism and extends lifespan
Source: Aging Cell. 2017 Jun 14;16(5):994–1005. doi: 10.1111/acel.12623 (PMC5595670; doi:10.1111/acel.12623)
Supplement: Supplementary file 4 — Appendix S1 Experimental procedures. [file ACEL-16-994-s004.docx]

**Additional Experimental Procedures**

*Inactivating mutations in ChES*

By using classical cloning techniques, we introduced inactivating mutations using the original plasmids with active full length chaperones as a starting point (Sambrook *et al.*, 1989). The schematic presentation of the domain architecture of each studied chaperone and the introduced mutations are presented in Fig. S2. The schemes of the domain architecture were generated using BLAST with default parameters (Boratyn *et al.*, 2012). In the case of Ssc1, the C-terminal domain essential for the translocation function of this chaperone was deleted (Blamowska *et al.*, 2010). In order to inactivate Lhs1, a point mutation was introduced at aspartate 26 into alanine by site directed mutagenesis, thus abolishing ATP binding capacity of the chaperone (de Keyzer *et al.*, 2009). In the case of Egd2 inactivation, the NAC domain (amino acids 17-72) was deleted (Ott *el al.*, 2015). To inactivate Tcp1, ATP binding capacity was abolished by deleting a portion of the ATP-magnesium binding site, namely LGPV motif (amino acids 44 to 47) (Brackley & Grantham, 2009). Cloning was performed in E. coli XL1 Blue (Stratagene).

*Growth curves*

The growth curves for the wild type, wild type bearing an empty vector, as well as each chaperone-enriched strain (ChES) were determined using the following procedure: overnight cultures were diluted 100 times in appropriate medium and optical density at 600 nm was measured every 30 minutes until saturation.

*Overexpression level measurement*

The expression level of each chaperone in the chaperone enriched strains was measured by using a rabbit polyclonal anti-His tag antibody (Abcam, ab137839, 1:10000) and secondary IgG goat anti-rabbit labeled with Alexa 488 (Thermo Fisher Scientific, A11034, 1:2000). The signal obtained by flow cytometry (mean fluorescence over 10000 cells) was compared to the endogenous expression level of each chaperone. Endogenous expression level was estimated by using flow cytometry measurement based on the GFP signal for Lhs1 and Egd2, for which GFP fusions were obtained from Thermo Scientific. The mean fluorescence intensity in ChES was normalized to the mean fluorescence intensity detected in wild type cells with endogenous expression of each chaperone. To determine the endogenous expression level of Ssc1 and Tcp1, the following primary antibodies were used: a rabbit monoclonal anti-Ssc1 (courtesy of Sven Dennerlein, 1:5000) and a rabbit monoclonal anti-Tcp1 (Abcam, ab92587, 1:1000) were used, followed by secondary IgG goat anti-rabbit labeled with Alexa 488 (Thermo Fisher Scientific, A11034, 1:2000) incubation and flow cytometry measurement. For yeast fixation, 5 mL of 37% formaldehyde was added directly to 45 mL of exponential culture, was mixed thoroughly, and was left to incubate for 45 min at room temperature. Cells were then collected by centrifugation at 1600xg for 4 minutes, and washed twice with 1 mL of ice cold fixation buffer (1.2M Sorbitol, 0.1 M potassium phosphate dibasic, pH 7.5). Cells were then resuspended in 1 mL fixation buffer containing 2.5µL zymolyase and were incubated for 1h at 30°C. After digestion, cells were spun down at 400xg for 5 minutes, and washed twice with ice cold fixation buffer. For permeabilization, cells were resuspended in 1mL 70% ethanol, and stored over night at 4°C. For blocking, 5% BSA in 10 mM PBS was used for 30 minutes. Primary and secondary antibodies were diluted in blocking buffer and incubated for 1h at room temperature, with 3 washes before and after the secondary antibody incubation. Then, flow cytometry was performed as described in the separate paragraph.

*Replicative lifespan measurement*

Replicative lifespan (RLS) for all strains was determined by micromanipulation in two ways: (i) with monitored cells constantly at 30^o^C, ad (ii) with overnight storage of monitored cells at 4^o^C. RLS measurement involves counting the number of daughters produced by individual mother cells. Using a microdissection apparatus equipped microscope suitable for yeast (Singer Instruments), cells were transferred to defined places on the agar plates and virgin daughter cells were collected. In case of (i), each cell was monitored continuously over several days every 60 - 90 min until all mother cells stopped budding. Total number of daughter cells was noted for each mother cell. In case of (ii), each cells was monitored continuously at 30^o^C during the day and the plate was stored at 4^o^C overnight to prevent the cells from budding. Cells were incubated on YPD (WT) or -URA (mutants) plates for the duration of the experiment. Each RLS measurement represents data pooled from 3 independent experiments. The total number of cells employed for each strain are as follows: 103 (wild type with empty plasmid), 120 (oe SSC1), 112 (oe LHS1), 129 (oe EGD2) and 127 (oe TCP1); 95 (ΔSnf1), 79 (ΔSnf1+LHS1), 95 (ΔSnf1+EGD2); 94 (caTor1), 89 (caTor1+LHS1), 94 (caTor1+EGD2); 100 (petite), 97 (petite+LHS1), 99 (petite+EGD2); 97 (wild type with an empty plasmid with overnight at 4^o^C), 109 (oe LHS1, overnight at 4^o^C), and 112 (oe EGD2, overnight at 4^o^C). The late life stage of the RLS was determined as the fraction of lifespan the one following the exponential decline of the survival. The survival curves were compared by using the log-rank (Mantel-Cox) test.

*Protein extraction and carbonylation measurement*

Cells were collected, washed and lysed in 10 mM PBS supplemented with zymolyase (0.06 U/μL), and protease inhibitor cocktail (Sigma). 10 μg/mL of the lipid removal agent (Sigma) was added to the lysates, incubated at RT for 1 h with shaking, and removed by centrifugation. Carbonylation measurement was performed as previously described (Wehr & Levine, 2013).

*Western blot detection of Hsp82, total Snf1, phospho-Snf1, total Sch9 and phospho-Sch9*

Cells were incubated in 1 mL lysis buffer (TBS, 60 U zymolyase, protease inhibitor cocktail) for 1 h at 37ºC and spheroplasts were collected after 5 min centrifugation at 3000 rpm. Pellet of spheroplasts was resuspended in spheroplast lysis buffer (1 mL buffer per 0.5 g of cell pellet; 0.6 M Sorbitol, 10 mM Tris-HCl pH 7.4, 1 mM PMSF). The pellet was vigorously vortexed for 1 min, and left on ice for 30 min, with occasional vortexing. After centrifugation, supernatant was collected, and protein concentration was measured using Bradford reagent (Sigma).

50 μg of protein was mixed with Laemmli sample buffer (10% SDS, 20% glycerol, 10 mM 2-mercaptoethanol, 0.05% bromophenol blue), heated to 95ºC for 5 min, and loaded onto two 7.5% SDS PAGE gels. One gel was then transferred onto a nitrocellulose membrane at 200 mA for 1 h while the second one was subjected to Coomassie staining.

For the determination of Hsp90 chaperone, the membrane was blocked with 5% milk in PBS containing 0.1% Tween-20 for 1 h. Anti-Hsp90 antibody (1 mg/mL) (StressMarq Biosciences) was diluted 2500x in blocking buffer, and incubated overnight at 4ºC. Detection was done by horseradish peroxidase-conjugated goat anti-mouse IgG secondary antibody (2 mg/mL) (Abcam), diluted 20,000x in blocking buffer.

For determination of phosphorylated Snf1, the membrane was blocked with 5% BSA in TBS containing 0.1% Tween-20 for 1 h. After the membrane was washed with TBS-T, rabbit monoclonal phospho-AMPK α (40H9) antibody (Cell Signaling Technology, 2535S) was diluted 25,000x in blocking buffer, and incubated overnight at 4ºC. The bound antibodies were detected by horseradish peroxidase-conjugated anti-rabbit Ig secondary antibody, diluted 7000x in blocking buffer. The same protocol was used to determine the total Snf1. As primary antibody, AMPKα1 (Cell Signaling, 2795) was used.

For determination of phosphorylated Sch9, the membrane was blocked with 5% BSA in TBS containing 0.1% Tween-20 for 1 h at room temperature. After the membrane was washed with TBS-T, rabbit anti-phospho-Sch9 antibody (courtesy of Robbie Loewith) was diluted 2500x in blocking buffer, and incubated overnight at 4ºC. The bound antibodies were detected by horseradish peroxidase-conjugated anti-rabbit Ig secondary antibody, diluted 5000x in blocking buffer. The same protocol was followed to determine the level of non-phosphorylated Sch9, only rabbit polyclonal anti-Sch9 was used as primary antibody (Abcam, ab56203).

Protein amount in a band of interest was quantified by using ImageJ software: intensity of the band was normalized to the total protein amount, i.e. the intensity of the entire lane of the Coomassie-stained gel, run simultaneously (described above).

*Microscopy: slide preparation*

Microscope slides were prepared as follows: 150μL of YPD or –Ura media containing 2% agarose was placed on a preheated microscope slide, and cooled, before applying yeast cells to obtain a monolayer. The cells were previously centrifuged at 4000xg for 3 min, and resuspended in 50μL YPD. Once dry, cover slip was placed and sealed.

### *Live cell imaging and image analysis*

Regardless of the object imaged, the same general setup was used. The slide was mounted on the Volocity software (version 6.3; Perkin Elmer) driven, temperature-controlled Nikon Ti-E Eclipse inverted/UltraVIEW VoX (Perkin Elmer) spinning disc confocal setup. Images were recorded through 60xCFI PlanApo VC oil objective (NA 1.4) using coherent solid state 488 nm/50 mW diode laser with DPSS module, and 1000x1000 pixels 14 bit Hamamatsu (C9100-50) electron-multiplied, charge-coupled device (EMCCD). The exposure time was 150 ms, and 5-10% laser intensity was used. The images were analyzed by using Image J software.

To measure the propensity of protein to aggregate in the control, ChES and iChES strains, the number of cells with at least one Hsp104-GFP foci was counted manually. More than 1000 cells was curated for each strain.

To visualize the morphology of the mitochondria in the control strain and the ChES, MitoLoc plasmid methodology was employed (courtesy of Markus Ralser) (Vowinckel *et al.*, 2015). Each studied yeast strain was transformed according to the described protocol (Gietz & Schiestl, 2007) with the only difference that the cells were incubated with the plasmid overnight at room temperature.

*Respiration measurement*

Oxygen uptake was monitored polarographically with an oxygraph equipped with a Clark-type electrode (Oxygraph, Hansatech, Norfolk, UK). Cells were harvested during exponential growth phase, spun and resuspended in appropriate medium at the density of 30 x 10^6^ cells/mL. 500 μL of culture were transferred to an airtight 1.5 mL oxygraph chamber. Cells were assayed in conditions closely similar to the ones in a flask culture (30°C and stirring). Oxygen content was monitored for at least 4 min. To ensure the observed oxygen consumption was due to the mitochondrial activity, complex III inhibitor antimycin (final concentration 10 μg/mL) was routinely added to the cultures and compared to the rate observed without antimycin.

*Geldanamycin and torin treatments*

The wild type cells and those of constitutively active Tor1 (caTor1), at diauxic shift, were exposed to 9 mM geldanamycin and incubated for the duration of one generation time, at 30^o^C. Also, the ΔSnf1 cells at the diauxic shift were exposed to 100 nm final concentration of torin and incubated for 2 hours before harvesting. The cells were then washed and subjected to RNA isolation and the measurement of oxygen consumption, as described elsewhere.

*Flow cytometry*

Flow cytometry was carried out on a Becton-Dickinson FACSCalibur model equipped with a 488 nm Argon laser and a 635 nm red diode laser.

*ROS measurement*

Cells were incubated in the dark with 10 μg/μL 2’,7’-dichlorofluorescein diacetate (H2DCFDA, Sigma) for 120 min at 37°C and subsequently analyzed on Becton-Dickinson FACSCalibur flow cytometer equipped with a 488 nm Argon laser and a 635 nm red diode laser. The fluorescence of 10,000 cells resulting from the intracellular conversion of non-fluorescent H2DCFDA into fluorescent 2’,7 -’dichlorofluorescein (DCF) was measured in FL1 channel. The collected data was analyzed using FlowJo software version 7.2.5 for Microsoft (TreeStar, San Carlos, CA, USA) to determine the mean green fluorescence intensity after each treatment. The results are expressed as the mean fluorescence of the 10,000 cells.

*Measurement of Mitochondrial Mass by Estimating Cardiolipin Content*

As a measure of mitochondrial mass, we used NAO, a dye that binds to cardiolipin, a phospholipid specifically present on the mitochondrial membrane (Petit *et al*, 1996; Rodriguez *et al*, 2008). The cells (1 x 10^6^ /mL) were incubated in 1 mL culture medium containing 100 nM NAO for 30 min in the dark at 30°C with constant shaking, followed by analysis on FACSCalibur flow cytometer with the same photomultiplier settings as used for DiOC6(3).

As a negative control, in each experiment, in aliquots of cells, the collapse of the mitochondrial membrane potential was achieved by preincubation with FCCP and antimycin at 100 μM and 5 μg/mL, respectively, 10 min before fluorescent dye staining.

*Assessment of Mitochondrial Membrane Potential*

Variations of the mitochondrial transmembrane potential (ΔΨm) were studied using DiOC6(3). Cells (1 x 10^6^ /mL) were incubated in 1 mL culture medium containing 40nM DiOC6(3) for 30 min in the dark at 30°C with constant shaking. DiOC6(3) membrane potential-related fluorescence was recorded using FL1-height. A total of 10,000 cells were analyzed for each curve. The collected data was analyzed using FlowJo software version 7.2.5 to determine the mean green fluorescence intensity after each treatment. The results are expressed as % of mean fluorescence of the control strain. For negative control, in each experiment, aliquots of cells were used to achieve the collapse of the mitochondrial membrane potential by preincubation with carbonyl-cyanide 4-(trifluoromethoxy)- phenylhydrazone (FCCP, Sigma) and antimycin (Sigma) at 100 μM and 5 μg/mL, respectively, 10 min before fluorescent dye staining.

*Statistical analysis*

Statistical analysis of data was performed using R v2.15.3 (*CRAN,* [*http://cran.r-project.org*](http://cran.r-project.org/)) and RStudio for Windows, v 0.97 ([*http://www.rstudio.com/*](http://www.rstudio.com/)). All groups were tested for normality of distribution using Shapiro-Wilk test. Since data followed normal distribution, the differences between multiple groups were compared using parametric one-way ANOVA, followed by Tukey’s *post-hoc* test. The differences between two groups were tested using Student’s two-tailed t-test. For all tests significance level was set at p<0.05.

*RNA extraction*

Total RNA was isolated from yeast cells following the procedure of the NucleoSpin RNA kit (Macherey&Nagel) for up to 3 x 10^8^ yeast cells, which dictates incubation with 50 - 100 U of zymolyase for 1 h at 30°C. The quality of resulting total mRNA was tested on 1% agarose gels.

*Quantitative real-time PCR*

cDNA was synthesized from 1000 ng of total RNA using iScript^TM^ cDNA Synthesis Kit (BioRad). The resulting cDNA was diluted 100 x, mixed with primer pairs for each gene and SYBRgreen (BioRad). The names of the genes whose expression was measured by qPCR as well as the sequences of the primers are shown in Table S2 (Supporting information). All primer pairs were designed to have a melting temperature of 60°C. The qPCR reaction was run on a QuantFlexStudio 6 (Life Technologies) using 40 cycles, after which the melting curves for each well were determined. qPCR differential expression was estimated from three- and four-fold replicates with EasyqpcR (Le Pape, 2015) by first removing Ct values over 1 standard deviation from the mean Ct for each gene/strain combination. Final fold change values were estimated relative to the UBC6 gene in the control strain replicates.

*RNA Sequencing*

RNA sequencing was performed for biological triplicates of each strain.

*Sequence mapping*

Sequenced reads (Illumina 50bp single-end) were mapped to the SacCer3 reference genome (*April 2001 revision,* [*http://www.ncbi.nlm.nih.gov/assembly/285498/*](http://www.ncbi.nlm.nih.gov/assembly/285498/)*, genome obtained from the UCSC Genome Browser; 25*) using the STAR aligner (*version 2.3.0e, 26*). Reference transcripts are obtained from the Ensembl yeast genome resource in GTF format.

*Read counting*

Raw read counts were determined from the overlaps with the Ensembl yeast genes using the htseq-count version 0.6.0. from the HTSeq framework (Anders *et al.*, 2014), with the following parameters: -a 10 –s no.

*Differential expression analysis*

Raw reads were analyzed for differential expression with the DeSeq2 package (Love *et al.*, 2014) within the Bioconductor framework (Huber *et al.*, 2015) under R statistical package (R Core Team, 2012), by previously filtering out the outlier replicates with PCA analysis on rlog transformed replicate data (Fig. S7).

*BN-PAGE and in-gel activity staining*

Mitochondria were isolated as previously described (Meisinger *et al.*, 2006). Briefly, mitochondria were solubilized in 1% digitonin, 20 mM Tris/HCl (pH 7.4), 5 mM EDTA, 100 mM NaCl, 10% (w/v) glycerol, and 2 mM PMSF to a final concentration of 1 mg/mL for 30 min at 4°C. Lysates were cleared by centrifugation (20000 g, 15 min, 4 °C) before addition of 10x loading dye (5% Coomassie brilliant blue G-250, 500 mM 6-aminohexanoic acid, 100 mM Bis-Tris, pH 7.0) and separated on 4%–13% polyacrylamide gradient gels with 4% stacking gel. Activity staining of respiratory chain complexes was performed at 30°C, according to published procedures (Wittig *et al.*, 2007). Briefly, for complex IV staining, gel stripes were incubated in 50 mM KPi (pH 7.4), 0.5 mg/mL diaminobenzidine and 1 mg/mL reduced cytochrome *c*. Complex V staining was performed in 35 mM Tris/HCl, 220 mM glycine (pH 8.3), 8 mM ATP, 14 mM MgSO_4_, 0.2% Pb(NO_3_)_2_.

*Metabolic labeling of newly synthesized proteins with L-AHA*

This step was performed using the Click-iT® metabolic labeling and detection of newly synthesized proteins kit (Invitrogen) according to the manufacturer's instructions. Briefly, 6.4 × 10^7^ cells are required to prepare a lysate with approximately 200 μg of protein. The Click-iT® metabolic labeling reagent was solubilized with DMSO to a final 1000x stock solution to ensure that the DMSO concentration is not more than 0.1–0.2% in the culture. Cells were washed once with warm PBS and methionine-free medium was added to the cells and incubated at 30°C for 90 min to deplete the cellular methionine reserves. Metabolic labeling reagent- L-AHA was added to the cell medium to the 40 µM final concentration, gently mixed, and incubated at 30°C for 90 min. Cells were pelleted by centrifugation at 400 g, 5 min. Cell pellet was washed 3 times with PBS, resuspended in lysis buffer (0.2 U/µl zymolyase, PBS, and protease inhibitors), and incubated at 37°C for 60 min. To solubilize the proteins and disperse the DNA, lysate was sonicated with a probe sonicator, vortexed for 5 min, then centrifuged at 10000 g at 4°C for 5 min. Protein concentration was measured using Bradford assay (Sigma). The protein samples were loaded onto two equivalent 1D SDS-PAGE gel (10% resolving gel): one was transferred onto a PVDF membrane and the second one stained by silver staining.

The detection was accomplished via a biotin alkyne detection molecule using the Click-IT ™ protein analysis detection kit (Invitrogen) and streptavidin-HRP on photographic film. Protein amount representing newly synthetized proteins was quantified by using ImageJ software: intensity of the each entire lane from each strain was normalized to the total protein amount, i.e. the intensity of the entire lane of the silver-stained gel, run simultaneously (described above).

**Supporting references:**

Anders S, Pyl PT, Huber W (2014) HTSeq—a Python framework to work with high-throughput sequencing data. *Bioinformatics* **31**, 166-169.

Boratyn GM, Schaefer AA, Agarwala R, Altschul SF, Lipman DJ, Madden TL (2012) Domain enhanced lookup time accelerated BLAST. *Biology Direct* **7**, 12.

Blamowska M, Sichting M, Mapa K, Mokranjac D, Neupert W, Hell K (2010) ATPase domain and interdomain linker play a key role in aggregation of mitochondrial Hsp70 chaperone Ssc1. *J. Biol. Chem.* **285**, 4423-4431.

Brackley KI, Grantham J (2009) Activities of the chaperonin containing TCP-1 (CCT): implications for cell cycle progression and cytoskeletal organisation. *Cell Stress Chaperones* **14**, 23-31.

De Keyzer J, Steel GJ, Hale SJ, Humphries D, Stirling CJ (2009) Nucleotide Binding by Lhs1p Is Essential for Its Nucleotide Exchange Activity and for Function in Vivo. J. Biol. Chem. **284**, 31564-31571.

Gietz D R, Schiestl R H (2007) High-efficiency yeast transformation using the LiAc/SS carrier DNA/PEG method*.* *Nat. Protoc.* **2**, 31–34.

Huber W, Carey VJ, Gentleman R, Anders S, Carlson M, Carvalho BS, Bravo HC, Davis S, Gatto L, Girke T, Gottardo R, Hahne F, Hansen KD, Irizarry RA, Lawrence M, Love MI, MacDonald J, Obenchain V, Oleś AK, Pagès H, Reyes A, Shannon P, Smyth GK, Tenenbaum D, Waldron L, Morgan M (2015) Orchestrating high-throughput genomic analysis with Bioconductor. *Nat. Methods* **12**, 115-121.

Love MI, Huber W, Anders S (2014) Moderated estimation of fold change and dispersion for RNA-seq data with DESeq2. *Genome Biol.* **15**, 550.

Meisinger C, Pfanner N, Truscott KN (2006) Isolation of yeast mitochondria. *Methods Mol. Biol.* **313**, 33-39.

#### Ott AK, Locher L, Koch M, Deuerling E (2015) Functional Dissection of the Nascent Polypeptide-Associated Complex in *Saccharomyces cerevisiae*. *PLoS One* 10, e0143457.

Petit P, Glab N, Marie D, Kieffer H, Métézeau P (1996) Discrimination of respiratory dysfunction in yeast mutants by confocal microscopy, image, and flow cytometry. *Cytometry* **23**, 28-38.

R Core Team (2012) R: A language and environment for statistical computing. R Foundation for Statistical Computing, Vienna, Austria. ISBN 3-900051-07-0.

Rodriguez ME, Azizuddin K, Zhang P, Chiu SM, Lam M, Kenney ME, Burda C, Oleinick NL (2008) Targeting of mitochondria by 10-N-alkyl acridine orange analogues: role of alkyl chain length in determining cellular uptake and localization. *Mitochondrion* **8**, 237-246.

Sambrook J, Fritsch EF, Maniatis T (1989) Molecular Cloning: A Laboratory Manual, 2nd Ed., Cold Spring Habor Laboratory Press, Cold Spring Harbor, NY

Vowinckel J, Hartl J, Butler R, Ralser M (2015) MitoLoc: A method for the simultaneous quantification of mitochondrial network morphology and membrane potential in single cells*.* *Mitochondrion* **24**, 77–86.

Wehr NB, Levine RL (2013) Quantification of protein carbonylation. *Methods Mol. Biol.* **965**, 265-281.

Wittig I, Karas M, Schaegger H (2007) High resolution clear native electrophoresis for in-gel functional assays and fluorescence studies of membrane protein complexes. *Mol. Cell Proteomics* **6**, 1215-1225.

**Supporting Information Figures:**

**Figure S1. Enrichment in four different chaperones from different cellular compartments results in alleviation of protein stress.**

**(A)** Flow cytometry based detection of the protein level in each ChES strain as well as in Hsp82 enriched strain was performed on fixed cells using an appropriate combination of primary and secondary antibody (for details see Methods). Control is wild type yeast with an empty vector. Data on the graph are mean ± SD from at two independent cultures. P values were calculated using ANOVA plus post hoc. *** p<0.001; ** p<0.01; * p<0.05.

**(B)** Western blot is shown revealing that ChES display a decreased level of Hsp90 relative to the control (wild type yeast with an empty vector). ß-actin band (loading control) was used for normalization.

**(C)** The fraction of cells containing at least one Hsp104-dependent protein aggregate is increased in iChES, strains enriched in inactivated versions of the studied chaperones. The protein aggregates were visualized via fusion of Hsp104 chaperone with GFP using the spinning disc confocal microscopy. Protein aggregation propensity is expressed as the fraction of cells with at least one aggregate. More than 1000 cells were screened for aggregates starting from two independent exponential yeast cultures for each strain. Control is wild type yeast with an empty vector. Data on the graph are mean ± SD from at two independent cultures. P values were calculated using ANOVA plus post hoc. *** p<0.001; ** p<0.01; * p<0.05.

**(D)** Protein carbonylation (PC), as a marker of protein oxidative damage, is slightly increased in iChES. ELISA based assay is used to measure the total amount of PC, where the absorbance at 450 nm reports on the PC level. Control is wild type yeast with an empty vector. Data on the graph are mean ± SD from at least 3 independent cultures, each performed in technical triplicate. P values were calculated using ANOVA plus post hoc. *** p<0.001; ** p<0.01; * p<0.05.

**(E)** The level of reactive oxygen species (ROS) is decreased in the ChES. Data are represented as mean ± SD from 3 independent cultures, each measured in duplicate. ** p<0.01 (ANOVA plus post hoc).

**(F)** The differential gene expression, measured by qPCR, characteristic of enrichment in inactivated versions of the studied chaperones does not phenocopy glucose starvation response. Top panel displays genes encoding respiratory chain components, and bottom panel the genes encoding metabolic enzymes as well as some typical stress markers. Control is wild type yeast with an empty vector. UBC6 was used for normalization. Data on the graph are mean ± SD from at least 3 independent cultures, each performed in technical triplicate. P values were calculated using ANOVA plus post hoc. *** p<0.001; ** p<0.01; * p<0.05.

**Figure S2. Examples of representative images of Hsp104-GFP tagged protein aggregates.** The imaging was performed using spinning disk confocal microscopy.

**Figure S3.** **The schematic presentation of domain architecture of each studied chaperone.** The images were generated using BLAST search against the non-redundant database, with each chaperone sequence as input. The parts of proteins deleted or modified by a point mutation are labeled with a purple rectangle or arrow.

**Figure S4. Chaperone activity is critical for the induction of the glucose starvation-like response.**

**(A)** Growth curves, measured as a growth in optical density of each strain at optimal growth temperature, 30^o^C, over time. The point at which the cells were harvested is labeled with an arrowhead for each strain. A representative curve is displayed for all four ChES since they display consistent results.

**(B)** Oxygen consumption does not change upon enrichment in inactivated versions of each chaperone (iChES). Control is wild type yeast with an empty vector. Data are mean ± SD from at least 3 independent cultures, each performed in triplicate. *** p<0.001; ** p<0.01; * p<0.05 (ANOVA plus post hoc).

**(C)** Mean mitochondrial volume was quantified using ImageJ MitoLoc plugin. As input, images obtained using spinning disc confocal microscopy were used, where each cell was analyzed individually for mitochondrial volume. More than 300 cells were screened for each strain. Data are mean ± SD from two independent cultures. *** p<0.001; ** p<0.01; * p<0.05 (ANOVA plus post hoc).

**(D)** The composition (Coomassie stain) and the activity *in vitro* of the respiratory chain complexes do not display any changes in the ChES relative to the control strain (wild type yeast with empty vector).

**Figure S5. TOR deactivation and consequent Snf1 activation are key events leading to the glucose starvation-like response in ChES.**

**(A)** Western blot detection of phosphorylated Snf1 and the ß-actin (loading control), used for normalization.

**(B)** Transcript levels of target genes in the case of chaperone enrichment in the absence of Snf1. Top panel displays genes encoding respiratory chain components, and bottom panel the genes encoding metabolic enzymes. Control is wild type yeast with an empty vector. UBC6 was used for normalization. Data on the graph are mean ± SD from at least 3 independent cultures, each performed in technical triplicate. P values were calculated using ANOVA plus post hoc. *** p<0.001; ** p<0.01; * p<0.05.

**(C)** Western blot based detection of the newly synthesized proteins and the corresponding total protein gel used for normalization in ChES strains, and in the strain with constitutively active Tor1 kinase (caTor1).

**(D)** Western blot based detection of Sch9 and phosphorylated Sch9. β-actin was used as loading control and the intensity of its band for normalization.

**(E)** Western blot detection of phosphorylated Snf1 and the ß-actin (loading control), used for normalization in strains with varying TOR pathway activity, and strains overexpressing each of the four studied chaperone in the genetic background of caTor1.

**(F)** Transcript levels of target genes in the case of chaperone enrichment in the background of constitutive Tor1 activity (caTor1). Control is wild type yeast with an empty vector was used. UBC6 was used for normalization. Data on the graph are mean ± SD from at least 3 independent cultures, each performed in technical triplicate. P values were calculated using ANOVA plus post hoc. *** p<0.001; ** p<0.01; * p<0.05.

**(G)** Transcript levels of target genes in the case of chaperone enrichment in the ΔTor1 background. Control is wild type yeast with an empty vector. Top panel displays genes encoding respiratory chain components, and bottom panel the genes encoding metabolic enzymes. UBC6 was used for normalization. Data on the graph are mean ± SD from at least 3 independent cultures, each performed in technical triplicate. P values were calculated using ANOVA plus post hoc. *** p<0.001; ** p<0.01; * p<0.05.

**(H)** Transcript levels of target genes in the ΔSnf1 treated with 100 nM torin. Control is wild type yeast with an empty vector. Top panel displays genes encoding respiratory chain components, and bottom panel the genes encoding metabolic enzymes. UBC6 was used for normalization. Data on the graph are mean ± SD from at least 3 independent cultures, each performed in technical triplicate. P values were calculated using ANOVA plus post hoc. *** p<0.001; ** p<0.01; * p<0.05.

**Figure S6. Hsp82 activity reduction results in Tor1 deactivation.**

**(A)** Western blot detection of phosphorylated Snf1 and the ß-actin (loading control), used for normalization in strains overexpressing Hsp82 together with each of the four studied chaperones. Control is wild type yeast with the empty plasmid.

**(B)** Western blot based detection of the newly synthesized proteins and the corresponding total protein gel used for normalization in strains overexpressing Hsp82 together with each of the four studied chaperones. Control is wild type yeast with the empty plasmid.

**(C)** Transcript levels of target genes upon treatment with geldanamycin in control (wild type yeast) and the caTor1 background. Left panel displays genes encoding respiratory chain components, and the right panel the genes encoding metabolic enzymes. UBC6 was used for normalization. Data on the graph are mean ± SD from at least 3 independent cultures, each performed in technical triplicate. P values were calculated using ANOVA plus post hoc. *** p<0.001; ** p<0.01; * p<0.05.

**Figure S7. (A) Replicative lifespan of ChES with overnight storage at 4^o^C.** The number of cells is 103, 97, 109, and 112 for the wild type with an empty plasmid continuously at 30^o^C, wild type with an empty plasmid with overnight at 4^o^C, oe LHS1 (overnight at 4^o^C), and oe EGD2 (overnight at 4^o^C), respectively.

Principle component analysis-based filtering of RNASeq replicates: First two principal component coordinates were used to identify outlier replicates and gave **(B)** PCA plot before removal of outliers, and **(C)** PCA plot after removal of outliers.

**Supporting Information Tables:**

**Table S1**. The summary of the RNA Sequencing results with the data for each strain organized in separate tabs.

**Table S2.** The list of genes with their transcript levels measured by qPCR in this study and used primers.
